# QRICH1 suppresses pediatric T-cell acute lymphoblastic leukemia by inhibiting GRP78

**DOI:** 10.1038/s41419-024-07040-7

**Published:** 2024-09-04

**Authors:** Ji’ou Zhao, Meiyun Kang, Huimin Li, Liucheng Rong, Yaping Wang, Yao Xue, Yuqian Yao, Yongjun Fang

**Affiliations:** Department of Hematology and Oncology, Children’s Hospital of Nanjing Medical University, Nanjing, Jiangsu Province China

**Keywords:** Acute lymphocytic leukaemia, Mechanisms of disease

## Abstract

T-cell acute lymphoblastic leukemia (T-ALL) is an aggressive hematological malignancy that commonly affects children and adolescents with a poor prognosis. The terminal unfolded protein response (UPR) is an emerging anti-cancer approach, although its role in pediatric T-ALL remains unclear. In our pediatric T-ALL cohort from different centers, a lower QRICH1 expression was found associated with a worse prognosis of pediatric T-ALL. Overexpression of QRICH1 significantly inhibited cell proliferation and stimulated apoptosis of T-ALL both in vitro and in vivo. Upregulation of QRICH1 significantly downregulated 78 KDa glucose-regulated protein (GRP78) and upregulated CHOP, thus activating the terminal UPR. Co-overexpression of GRP78 in T-ALL cells overexpressing QRICH1 partially reverted the inhibited proliferation and stimulated apoptosis. QRICH1 bound to the residues Asp212 and Glu155 of the nucleotide-binding domain (NBD) of GRP78, thereby inhibiting its ATP hydrolysis activity. In addition, QRICH1 was associated with endoplasmic reticulum (ER) stress in T-ALL, and overexpression of QRICH1 reversed drug resistance. Overall, low QRICH1 expression is an independent risk factor for a poor prognosis of pediatric T-ALL. By inhibiting GRP78, QRICH1 suppresses pediatric T-ALL.

## Introduction

T-cell acute lymphoblastic leukemia (T-ALL) accounts for ~15% of pediatric ALL cases, serving as one of the most aggressive hematological malignancies with a poor prognosis [1]. Hyperleukocytosis, extramedullary infiltration, and mediastinal mass are typical clinical features of pediatric T-ALL [2]. Although an early intensive therapy and targeted treatment provide survival benefits [3, 4], children with early T-cell precursor (ETP) leukemia, refractory or relapsed (R/R) T-ALL, and central nervous system leukemia (CNSL) suffer from an extremely poor prognosis [5–7]. Pediatric T-ALL has a relatively low event-free survival (EFS) but high risks of relapse and early death [8, 9]. Therefore, it is urgent to develop effective approaches against pediatric T-ALL.

Protein anabolism in the endoplasmic reticulum (ER) is an important component of cellular metabolism. However, production of unfolded or misfolded proteins during the protein processing in ER leads to ER stress (ERS), in which cells restore homeostasis by initiating the downstream unfolded protein responses (UPRs) [10]. There are three branches of the UPR, namely inositol-requiring enzyme 1α (IRE1α), protein kinase R-like endoplasmic reticulum kinase (PERK) and activating transcription factor 6 (ATF6) signaling pathways. IRE1α, PERK and ATF6 bind to a 78 KDa glucose-regulated protein (GRP78) in an inactivated state absent from ERS, but on the contrary, dissociate from GRP78 to activate the three branches in the presence of ERS [11]. An UPR can be adaptive or terminal. Adaptive UPR induced by a moderate ERS promotes cell survival by reducing protein loads and maintaining cell homeostasis. In contrast, terminal UPR predominates in high-strength or persistent ERS and causes cell death [12–14].

Both the extrinsic (hypoxia, nutrient deprivation, reactive oxygen species accumulation, and low pH) and intrinsic (chromosome number, genomic instability, and increased secretion) cancer factors cause ERS [10, 15]. Malignant cells are more dependent on the pro-survival pathway of adaptive UPR compared to normal cells [16]. Approaches to induce terminal UPR has become promising in killing malignant cells, although the way to convert adaptive UPR into terminal UPR is challenging [17, 18].

Glutamine-rich protein 1 (QRICH1) locates on chromosome 3p21.31, containing 11 exons and encoding a protein of 776 amino acids. Loss-of-function variants of QRICH1 are associated with the Ververi-Brady syndrome (VBS) manifested as intellectual disability, speech delay, and mild dysmorphic facial features [19, 20]. QRICH1 is involved in an increased protein toxicity under ERS and induction of terminal UPR [21]. In our study, we hypothesized that QRICH1 was able to inhibit pediatric T-ALL by inducing terminal UPR, and explored the exact molecular mechanism in in vitro and in vivo models.

## Materials and methods

### Clinical samples and raw data

Peripheral blood mononuclear cells (PBMCs) were separated from 3 patients with urogenital malformations in our hospital by density gradient centrifugation. CD3^+^ T lymphocytes were isolated from PBMC using CD3 magnetic beads (Anti-Human CD3 Magnetic Particles, No. 552593, BD Biosciences) as negative controls. Incipient and remission bone marrow samples from 3 children with T-ALL were used for protein extraction, and their baseline characteristics were listed in Supplementary Table 1. Transcriptomic data and clinical data of 23 T-ALL cases admitted in Children’s Hospital of Nanjing Medical University were used for statistical analysis. In addition, T-ALL samples available from the public datasets of GSE72623, GSE147930, GSE161895, GSE78220 and TARGET-ALL-Phase II project were extracted for analysis. Written informed consent was obtained from guardians of each participant before the study. This trial was approved by the Ethics Committee of the Children’s Hospital of Nanjing Medical University (No. 202404028-1).

### Cell culture and lentiviral transduction

The Jurkat (ZQXZBIO, Shanghai, China, No. CSP042) and HPBALL cell lines (Key Laboratory of Pu-Erh Tea Science, Yunan Agricultural University) were cultured in RPMI 1640 Medium containing 10% fetal bovine serum (FBS) in a cell incubator with 5% CO2 at 37 °C.

The lentivirus plasmid GV341 (Ubi-MCS-3FLAG-SV40-puromycin, Genechem, Shanghai, China, No. GOSL0369987) and the QRICH1 gene sequence were digested by AgeI and NheI restriction enzymes, and a complete cloning was conducted through the in-fusion recombination method. Recombinant vector was detected by DNA sequencing. For cell transfection, 1 × 10^5^ cells with 2–5 × 10^6^ TU of virus per well were implanted in 24-well plates. Fresh medium with 500 μL per well with 4 μg/μL puromycin was applied. The transfection efficiency was verified by qPCR and western blot. GRP78 overexpression lentivirus (GM-85763LV) and the control lentivirus (GM-6946LV, No. GM-LC-141426) were purchased from Genomeditech Co., Ltd (Shanghai, China). Amplified sequences were inserted into PGMLV-CMV-MCS-EF1-ZsGreen1-T2A-Blasticidin according to the manufacturer’s protocol. Blasticidin at a concentration of 7.5 μg/μL was used for screening after transfection.

### Functional experiments in vitro

The cell proliferation was tested using the EdU assay kit (RiboBio, No. C10310-1) and cell counting kit-8 (CCK-8)(APExBIO, No. K1018). Cell apoptosis and cell cycle progression were determined by Annexin V/7AAD staining (BD Biosciences, No. 559763) and propidium iodide (PI) staining (MULTI SCIENCES, No. 70-CCS012), respectively using flow cytometry (Beckman Coulter CytoFLEX) equipped with the FlowJo software. Cellular ATP level was measured using the ATP content assay kit (Nanjing Jiancheng Bioengineering Institute, No. A095-1-1). Cell viability was detected by SYTO13(Thermo Fisher Scientific-CN, No.1921359) staining.

### Animal experiments

A total of 19 female BALB/c nude mice (Animal Core Facility of Nanjing Medical University) were housed under specific pathogen-free (SPF) conditions, and randomly assigned into PBS group (*n* = 3), Vector1 group (*n* = 8) and QRICH1 group (*n* = 8). Tail vein injection of 200 µL of PBS, PBS containing 2 × 10^6^ HPBALL cells transfected with NC, and PBS containing 2 × 10^6^ HPBALL cells overexpressing QRICH1 was performed in the three groups, respectively. After 3 weeks of inoculation, 3 mice in each group were randomly sacrificed for sampling and experimental analysis. Engrafted human leukemia cells (hCD3^+^ cells) in the bone marrow and peripheral blood were detected by flow cytometry. Hematoxylin and eosin (H&E) and immunofluorescence staining were performed in paraffin-embedded spleen sections. The remaining nude mice were followed up for survival. Mouse livers were harvested after death and frozen in a −80 °C freezer for subsequent qPCR assays. The animal experiments were approved by the Laboratory Animal Welfare Ethics Committee of Nanjing Medical University (No. 2401020-1).

### Immunofluorescence analysis

The mouse spleen was extracted and fixed in 4% paraformaldehyde overnight, dehydrated in alcohol at a gradient concentration, transparent and dipped in paraffin. After paraffin embedding, 4 μm sections were prepared and baked in an oven at 60 °C. Tissue sections were then deparaffinized and rehydrated for antigen repair, and blocked with 3% BSA for 30 min. They were incubated with anti-GRP78 antibody (mouse, Proteintech, No. 66574-1-lg, 1:200) and anti-Ki67 antibody (rabbit, Huaabio, No. HA72115, 1:200) overnight at 4 °C. After washing with PBS for 3 times, sections were incubated with corresponding secondary antibodies for 50 min at room temperature in the dark. The tissue autofluorescence was quenched by DAPI repainting of cell nuclei, and sections were sealed using an anti-fluorescence quenching sealer. Image acquisition was performed using a laser confocal microscope (Zeiss LSM710, Germany).

### Liquid chromatography-tandem mass spectrometry (LC–MS/MS)

Protein supernatants of Jurkat and HPBALL cells mixed with a buffer were prepared for co-immunoprecipitation (IP) using an anti-QRICH1 antibody (Abcam, No. AB241574), followed by SDS-PAEG, Coomassie Brilliant Blue staining, gel cutting and tryptic digestion. Peptide samples were dissolved in mobile phase A (0.1% formic acid) and separated by EASY nLC-1200 (Thermo Scientific). Mass spectrometry (MS) in a data-dependent scan mode was performed, and 20 most abundant precursor ions of each MS were selected for MS/MS with a 20-sec dynamic exclusion. Mass spectra were processed and searched using Proteome Discoverer (version 2.4, Thermo Scientific) against the human Swissprot protein database (release 2022_01).

### Western blotting

Cells were lysed on ice for 15 min using protease inhibitor-containing cell lysis solution, and centrifuged at 13,000 rpm for 15 min. The supernatant was mixed with sample buffer, denatured at 95 °C for 10 min, and frozen at −20 °C. Protein samples were separated by SDS-PAGE and transferred to PDVF membranes (Bio-Rad, USA), followed by 2-hour immersing in 5% skimmed milk at room temperature. The membranes were then incubated with primary antibodies at 4 °C overnight and secondary antibodies at room temperature for 1 h. Protein bands were detected using chemiluminescent detection system (Bio-Rad, USA).

### Real-time quantitative PCR

Total RNA was extracted using TRIzol reagent (Invitrogen, No. 99090201), and reversely transcribed into cDNA using the 5×HiScripe II qRT superMix reagent (Vazyme, No.7E782C3). DNA amplification and quantification were conducted by the 2×AceQ qPCR SYBR Green Master Mix reagent (Vazyme, No.7E752D3) on a PCR system (Roche LightCycler 96, German). Data were calculated by the 2^-ΔΔCT^ method. The primer sequences were shown in Supplementary Table 2.

### Statistical analysis

Results of all experiments were presented as mean ± standard deviation of three independent experiments. The Student’s *t*‑test and ANOVA were used to assess the differences between the two groups and among three or more groups, respectively. GraphPad Prism 8.0 was used for statistical analysis and graphing, and R software was used for bioinformatics analysis. *P* < 0.05 was considered as a significant difference.

## Results

### Low QRICH1 expression is an independent risk factor for a poor prognosis of pediatric T-ALL

**Fig. 1:**
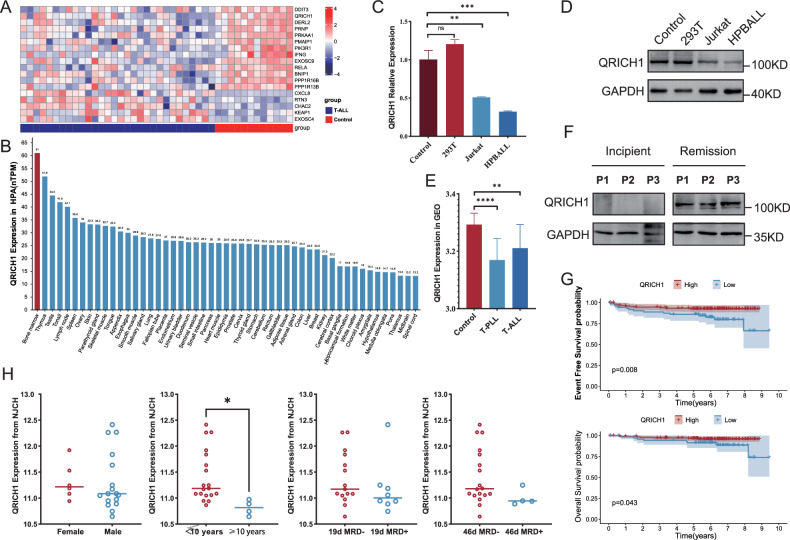
Expression level of QRICH1 and its prognostic significance. **A** RNA-seq screened 18 UPR-related differentially expressed genes between pediatric T-ALL cases and controls. **B** Expression levels of QRICH1 in different human tissues. Downregulated mRNA (**C**) and protein levels QRICH1 (**D**) in pediatric T-ALL cells. **E** Downregulated QRICH1 in pediatric T-ALL and adult T-PLL in the GEO database. **F** Downregulated QRICH1 in bone marrow samples of incipient pediatric T-ALL than remission samples. **G** Analysis of the TCGA database showed lower EFS and OS in pediatric T-ALL cases with low QRICH1 expression. **H** Low expression of QRICH1 in the high-risk subgroups of 23 T-ALL patients in our hospital.

To illustrate genes responsible for activating terminal UPR, we screened 352 ER-related genes with correlation coefficients≥3 from the GeneCard database (https://www.genecards.org/). Their differential expressions were analyzed in 30 pediatric T-ALL samples from the GSE72623 dataset and 12 normal human T-cell samples from the GSE147930 dataset. In total, 18 differentially expressed genes (DEGs) in pediatric T-ALL were obtained (Fig. 1A). Further searched in the Human Protein Atlas database (https://www.proteinatlas.org/), QRICH1 expression was the highest in normal bone marrow and thymus (Fig. 1B). ALL of these increased our confidence in studying it in pediatric T-ALL. In comparison to normal pediatric CD3^+^ T cells (negative control) and 293 T cells (tool cells as an auxiliary control), the mRNA and protein levels of QRICH1 were significantly lower in Jurkat and HPBALL cells (Fig. 1C, D). In the GSE72623 and GSE147930 datasets, QRICH1 was consistently downregulated in adult T-cell prolymphocytic leukemia (T-PLL) and pediatric T-ALL (Fig. 1E). We randomly collected paired bone marrow samples from three incipient and remission T-ALL patients, and QRICH1 was found significantly upregulated in all remission samples (Fig. 1F). To further clarify the clinical significance of QRICH1, 226 eligible patients (Supplementary Information 1) from the TARGET-ALL-Phase II Project were assigned into high QRICH1 expression and low QRICH1 expression groups. The survival analysis showed significantly lower long-term EFS and overall survival (OS) in the low QRICH1 expression (Fig. 1G). Then, propensity score matching (PSM) based on risk factors like gender, age, initial leukocyte count, central nervous system leukemia/ testicular leukemia (CNSL/TL) status, 29-day measurable residual disease (MRD), and biological characteristics with a caliper value of 0.02 were performed. Baseline characteristics of 226 patients from the TARGET-ALL-Phase II Project before and after matching were shown in Supplementary Tables 3 and 4. We obtained 40 pairs of exact matches, and the recurrent rate of T-ALL was significantly lower in the high QRICH1 expression group (1 recurrence) than the low QRICH1 expression group (8 recurrences). Further COX regression analysis based on survival status showed that low QRICH1 expression was an independent risk factor for a poor prognosis of pediatric T-ALL (Supplementary Table 5). Moreover, 23 pediatric cases of T-ALL from our hospital were stratified by gender, age (<10 years versus ≥10 years), 19-day MRD, and 46-day MRD. As expected, expression levels of QRICH1 were lower in all high-risk groups (Fig. 1H).

### QRICH1 suppresses pediatric T-ALL in vitro

**Fig. 2:**
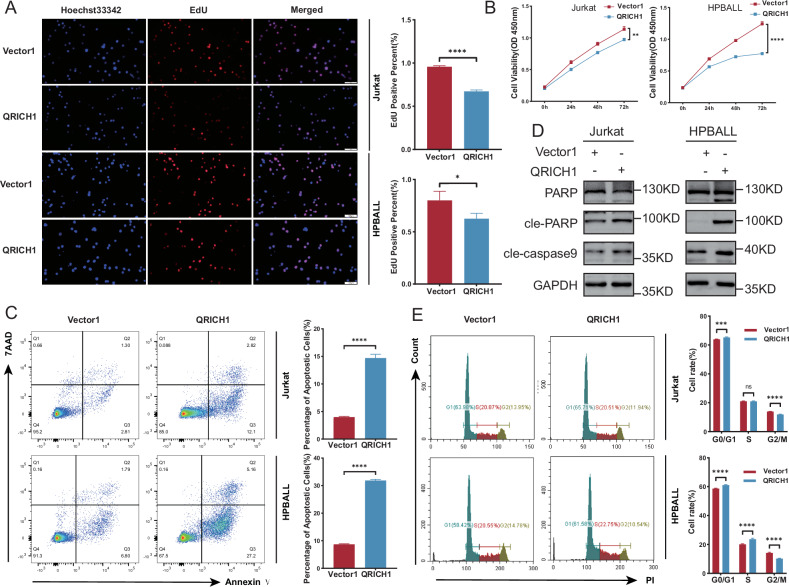
In vitro functional validation of QRICH1. **A** EdU assay showed decreased proliferation in cells overexpressing QRICH1. **B** CCK-8 assay from 3 replicate experiments showed a reduced cell viability in cells overexpressing QRICH1. **C** Annexin V/7AAD double staining showed an increased apoptosis in cells overexpressing QRICH1. **D** Downregulation of total PARP and upregulation of cle-PARP and cle-caspase9 in cells overexpressing QRICH1. **E** Flow cytometry showed an increased proportion of cells overexpressing QRICH1 in the G0/G1 phase and a decreased proportion in the G2/M phase.

To investigate the role of QRICH1 in pediatric T-ALL, Jurkat and HPBALL cell lines overexpressing QRICH1 were constructed by lentivirus transduction. Both EdU and CCK-8 assays showed significantly inhibited proliferation in Jurkat and HPBALL cells overexpressing QRICH1 (Fig. 2A, B). A significantly enhanced apoptotic rate was observed in T-ALL cells overexpressing QRICH1 than those of negative controls (Fig. 2C). Consistently, downregulation of full-length PARP and upregulation of cleaved PARP (cle-PARP) and cleaved caspase9 (cle-caspase9) after overexpression of QRICH1 were also suggestive of a stimulated apoptosis (Fig. 2D). We further examined cell cycle progression and found a significantly higher proportion of cells in the G0/G1 phase, but lower proportion of cells in the G2/M phase after QRICH1 overexpression (Fig. 2E), suggesting that QRICH1 overexpressing T-ALL cells were arrested at the early stage of the cell cycle.

### QRICH1 triggers terminal UPR via GRP78

**Fig. 3:**
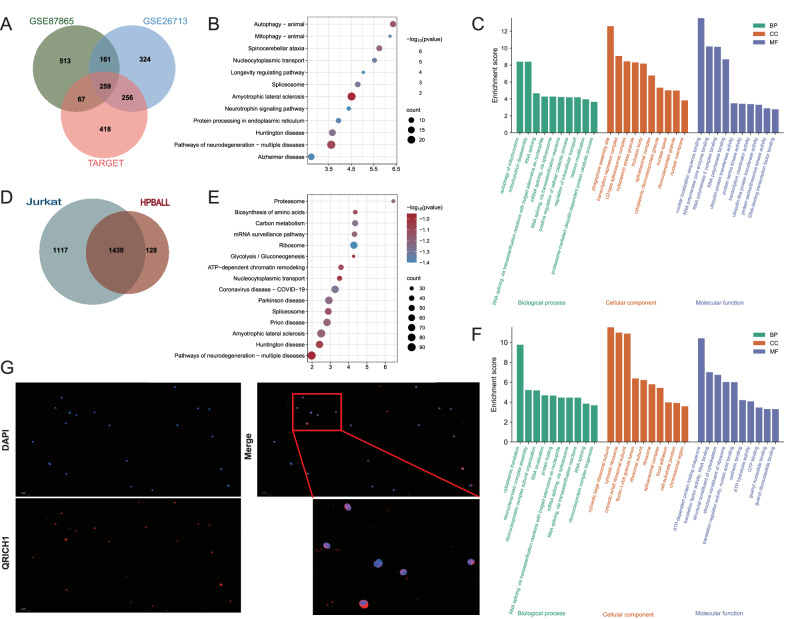
Functional clustering analysis of QRICH1. **A** An intersection of 3 pediatric T-ALL RNA-seq datasets yielded 259 genes highly correlated with QRICH1. **B**, **C** QRICH1 and the KEGG and GO enrichment analyses at the mRNA level. **D** 1,430 proteins binding to QRICH1 in Jurkat cells and HPBALL cells. **E**, **F** QRICH1 and the KEGG and GO enrichment analyses at the protein level. **G** Immunofluorescence staining of human peripheral blood showed the nuclear and cytoplasmic localization of QRICH1.

Through intersecting transcriptome data of T-ALL from three datasets (GSE87865, GSE26713, and TARGET-ALL-Phase II project), a set of 259 genes were highly correlated with QRICH1 (Fig. 3A). Gene Ontology (GO) and Kyoto Encyclopedia of Genes and Genomes (KEGG) enrichment analyses revealed that QRICH1 was enriched in autophagy, RNA splicing, protein processing, and neuroprotein disorders (e.g., protein processing in endoplasmic reticulum, mRNA splicing via spliceosome and pathways of neurodegeneration-multiple diseases) (Fig. 3B, C). Immunoprecipitation in Jurkat cells and HPBALL cells yielded a total of 1430 intersecting proteins enriched in the anti-QRICH1 antibody (Fig. 3D). Interestingly, they were also functionally involved in the biosynthesis of amino acids, Parkinson disease, RNA localization and protein folding (Fig. 3E, F). Gene Set Enrichment Analysis (GSEA) further revealed that QRICH1 was associated with the protein processing in endoplasmic reticulum pathway (Supplementary Fig. 1A). Immunofluorescence revealed that QRICH1 was localized in both the nucleus and cytoplasm, corresponding to its function of RNA and protein processing (Fig. 3G).

**Fig. 4:**
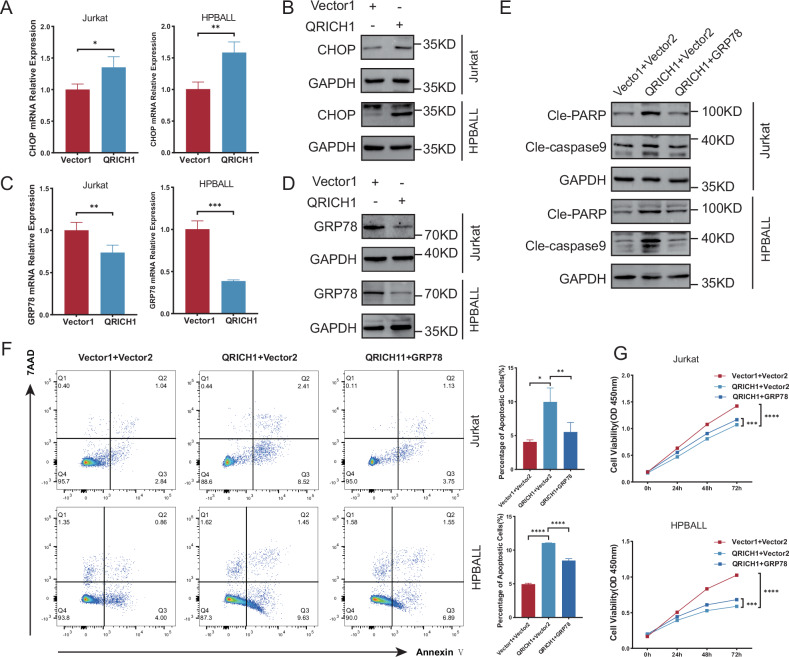
GRP78 is a downstream target of QRICH1. **A**, **B** Overexpression of QRICH1 upregulated the mRNA and protein levels of CHOP. **C**, **D** Overexpression of QRICH1 downregulated the mRNA and protein levels of GRP78. **E** Expression levels of apoptotic proteins in cells co-overexpressing GRP78 and QRICH1. **F** Flow cytometry showed apoptosis in cells co-overexpressing GRP78 and QRICH1. **G** CCK-8 assay showed that overexpressed GRP78 partially rescued cell viability in cells overexpressing QRICH1 from 3 replicated experiments.

Given that QRICH1 is involved in protein processing and can promote terminal UPR [21]. To further validate it, our experimental results showed that XBP1s(IRE1α pathway activation marker), PERK and ATF6, which were established regulators of UPR, were significantly upregulated in T-ALL cells overexpressing QRICH1 (Supplementary Fig. 1B). The transcription factor C/EBP homologous protein (CHOP) is their downstream to trigger terminal UPR by activating the apoptotic pathway [22]. Here, the protein and mRNA levels of CHOP were significantly elevated by overexpression of QRICH1 (Fig. 4A, B). GRP78 is an on/off switch for the UPR that binds to IRE1α, PERK, and ATF6 to maintain an inactivated state of UPR. The absence of GRP78 activates UPR via IRE1α, PERK, and ATF6 [23]. In our study, enrichment analyses have highlighted the involvement of GRP78 in the function of QRICH1. As shown in Fig. 4C, D, both the mRNA and protein levels of GRP78 were significantly reduced in T-ALL cells overexpressing QRICH1. Co-overexpression of GRP78 and QRICH1 showed that changes in cell apoptosis (Fig. 4E, F) and viability (Fig. 4G) caused by QRICH1 overexpression were partially rescued by overexpressed GRP78.

**Fig. 5:**
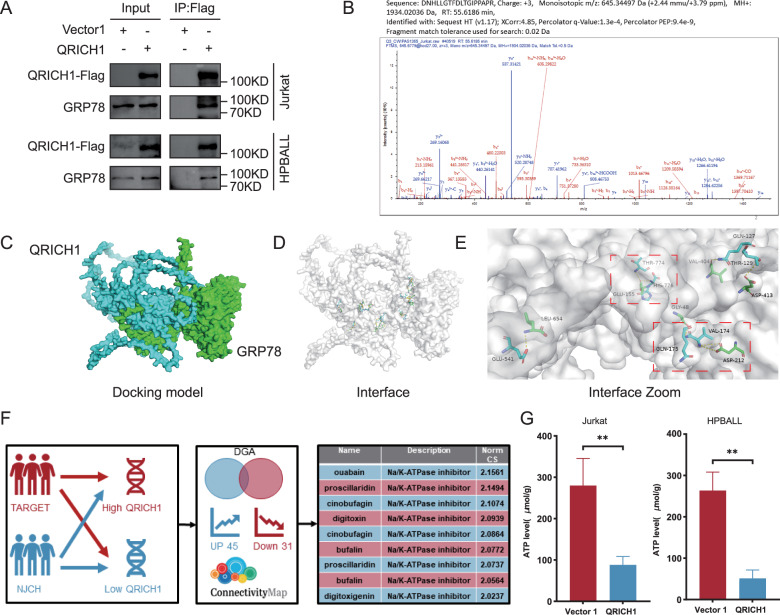
Interaction between QRICH1 and GRP78. **A** Immunoprecipitation demonstrated the binding of QRICH1 and GRP78. **B** Peptides corresponding to the QRICH1-binding protein GRP78 in Jurkat cells. **C** The optimal predictive model of QRICH1 binding to GRP78 with a binding energy of −720 kcal/mol. **D** A total of 17 pairs of amino acid residues form hydrogen bonds on the docking surface of QRICH1 and GRP78. **E** The pivotal binding sites Asp212 and Glu155 in the NBD of GRP78. **F** Connectivity mapping for molecular compounds with a positive correlation with QRICH1 bioactivity. **G** ATP content in Jurkat cells and HPBALL cells overexpressing QRICH1.

Co-IP confirmed an interaction between QRICH1 and GRP78 (Fig. 5A). LC–MS/MS consistently detected that GRP78 was pulled down by the anti-QRICH1 antibody (Fig. 5B and Supplementary Fig. 1C). Next, we performed rigid protein-protein docking between QRICH1 and GRP78 (https://gramm.compbio.ku.edu/), choosing the lowest binding energy model with a binding energy of −720 kcal/mol (Fig. 5C). A total of 17 pairs of amino acid residues were identified with hydrogen bonds between QRICH1 and GRP78 (Fig. 5D), of which, Asp212 and Glu155 were residues in the nucleotide-binding domain (NBD) of GRP78 (Fig. 5E). NBD allows ATP hydrolysis and provides energy for GRP78 binding to substrates, thus alleviating ERS [24]. Therefore, we believed that QRICH1 inhibited the ATPase activity of the NBD domain of GRP78 by binding to Asp212 and Glu155 residues. DEGs were detected between the high and low QRICH1 expression groups, consisting of 45 upregulated and 31 downregulated genes. Connectivity mapping (cMap, https://clue.io/) visualized the molecular compounds with a most positive correlation with QRICH1 bioactivity (Supplementary Table 6). Interestingly, most of them were ATPase inhibitors, including salermide, ouabain, and proscillaridin (Fig. 5F). ATP content was significantly reduced in cells overexpressing QRICH1, serving as a key event to affect the NBD of GRP78 and its function (Fig. 5G).

### QRICH1 suppresses T-ALL infiltration and proliferation in vivo

**Fig. 6:**
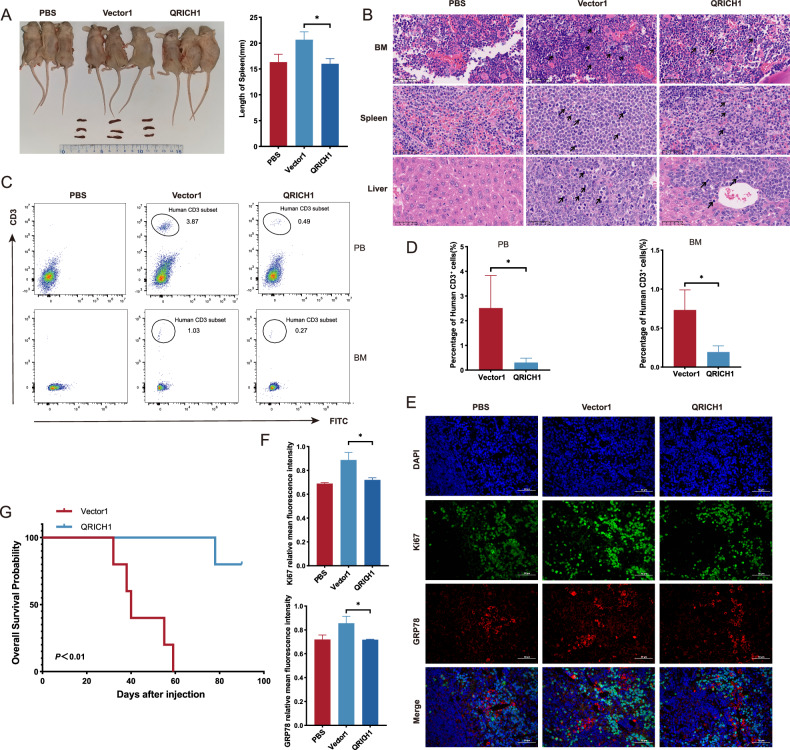
QRICH1 provides survival benefit to T-ALL mice. **A** A gross view of mouse spleen in the PBS group, Vector 1 group and QRICH1 group. **B** H&E staining of bone marrow, spleen and liver of mice 3 weeks after tail vein injection. **C**, **D** Flow cytometry of human CD3^+^ cells in peripheral blood and bone marrow of mice. **E**, **F** Immunofluorescence staining of mouse spleens to localize and quantify Ki67 and GRP78. **G** Kaplan–Meier curves comparing the survival of mice in the QRICH1 group and Vector1 group with a follow-up period of 90 days.

A T-ALL mouse model was established by tail vein injection of HPBALL cells. Three weeks after xenotransplantation, mice in the Vector1 group showed a significant wasting appearance, decreased daily activity and depression. Three randomly selected mice per group were sacrificed to harvest peripheral blood, bone marrow, spleen and liver samples. Mice in the Vector1 group showed the largest gross view of spleen than other groups (Fig. 6A). We further assessed the organ infiltration via H&E staining. As shown in Fig. 6B, significant infiltrations of leukemia cells in the bone marrow, spleen, and liver of T-ALL mice positive controls (Vector1 group) than those overexpressing QRICH1 suggested the protective effect of QRICH1 on T-ALL in vivo. Flow cytometry consistently revealed a significantly decreased percentage of hCD3^+^ cells in peripheral blood and bone marrow of T-ALL mice overexpressing QRICH1 compared to the Vector1 group (Fig. 6C, D). Immunofluorescence staining showed that the expression levels of nuclear Ki67 and cytoplasmic GRP78 were significantly higher in mouse spleen of the Vector1 group than the QRICH1 group (Fig. 6E, F). The remaining mice were followed up for 90 days. Interestingly, a longer survival was observed in mice of the QRICH1 group than other groups (*P* = 0.0018), and they remained alive until the end of follow-up (Fig. 6G). Finally, qPCR showed that the mRNA levels of Ki67 and PCNA in mouse liver were significantly lower in the QRICH1 group than those of Vector1 group (Supplementary Fig. 1D). Overall, we have proved that QRICH1 overexpression suppressed leukemia cell infiltration and proliferation in vivo.

### Persistent ERS increases QRICH1 expression and enhances terminal UPR

**Fig. 7:**
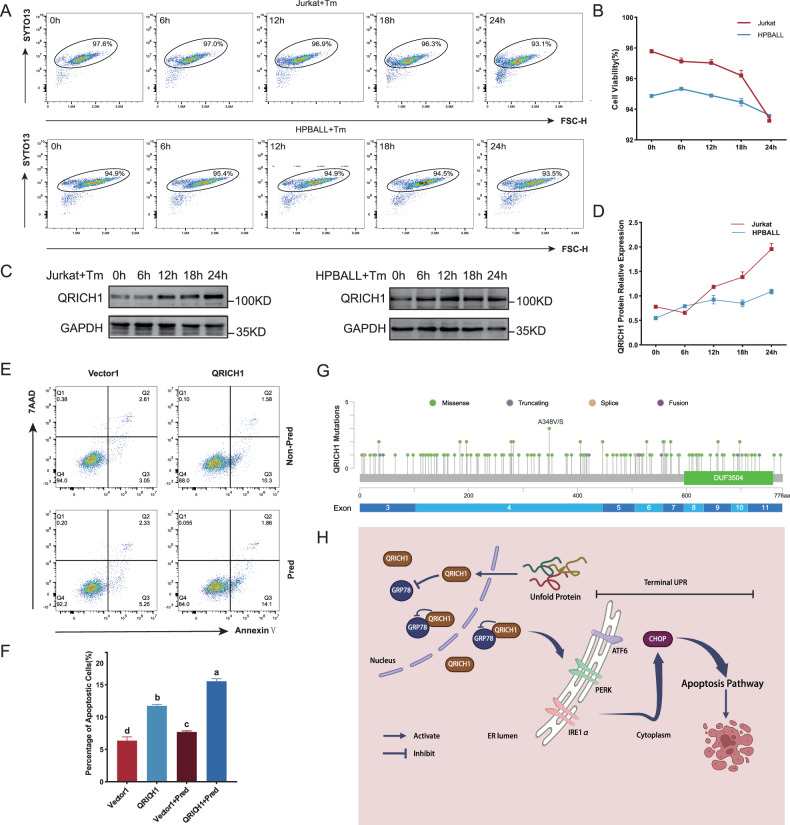
Regulation of QRICH1. **A**, **B** SYTO 13 staining was used to detect cell viability at different time points after 2.5 μM tunicamycin (Tm) induction. **C**, **D** Expression levels of QRICH1 at different time points after 2.5 μM Tm induction. **E**, **F** Apoptosis of prednisone-resistant cells overexpressing QRICH1. **G** Diagram of QRICH1 gene mutation sites in TCGA pan-cancer. **H** Unfolded proteins promote the expression of QRICH1 and then activates the terminal UPR pathway by binding to GRP78 and downregulating it.

To investigate how ERS influenced QRICH1, T-ALL cells were induced with tunicamycin (Tm), known as a classical inducer of ERS. It is well known that persistent ERS can induce terminal UPR. After a continuous incubation with 2.5 μM Tm [14] (Supplementary Fig. 1E), nuclear acid staining of Jurkat and HPBALL cells by SYTO 13 showed a decrease in cell viability within 24 h, which was more pronounced from 18 h to 24 h (Fig. 7A, B). Correspondingly, protein expression of QRICH1 was gradually upregulated in Tm-induced cells, especially in the period of 18 h to 24 h (Fig. 7C, D). It is indicated that QRICH1 responded to ERS, and may had a link with terminal UPR. We therefore hypothesized that endogenous QRICH1 produced under ERS coupled with exogenous QRICH1 enhances the pro-apoptotic effect. In fact, ERS-induced T-ALL cells overexpressing QRICH1 exhibited a significant synergistic effect (Supplementary Fig. 2A, B), which may be a vital clue for reversing chemotherapy resistance. In comparison to prednisone-resistant HPBALL cells [25], overexpression of QRICH1 significantly promoted cell apoptosis, suggesting a promising potential of QRICH1 in reversing chemotherapy resistance in T-ALL (Fig. 7E, F).

In addition, we analyzed QRICH1 gene mutations in the Cancer Genome Atlas (TCGA) PanCancer Atlas using the online tool cBioPortal [26]. A total of 133 mutation sites were identified, including 111 missense, 12 truncating, 4 splice and 6 fusion mutations (Fig. 7G and Supplementary Information 2). Among them, missense mutation and deep deletion were the most predominant mutation types. QRICH1 gene mutations were mainly detected in mature B-cell neoplasms, endometrial cancers and melanomas (Supplementary Fig. 2C). Interestingly, shallow deletion was dominant in the mRNA expression of QRICH1 in almost all types of cancers (Supplementary Fig. 2D).

Overall, we believed that ERS increased the expression level of QRICH1, thus activating the terminal UPR pathway by binding to GRP78 and downregulating its expression (Fig. 7H).

### QRICH1 involves in the tumor immunity

**Fig. 8:**
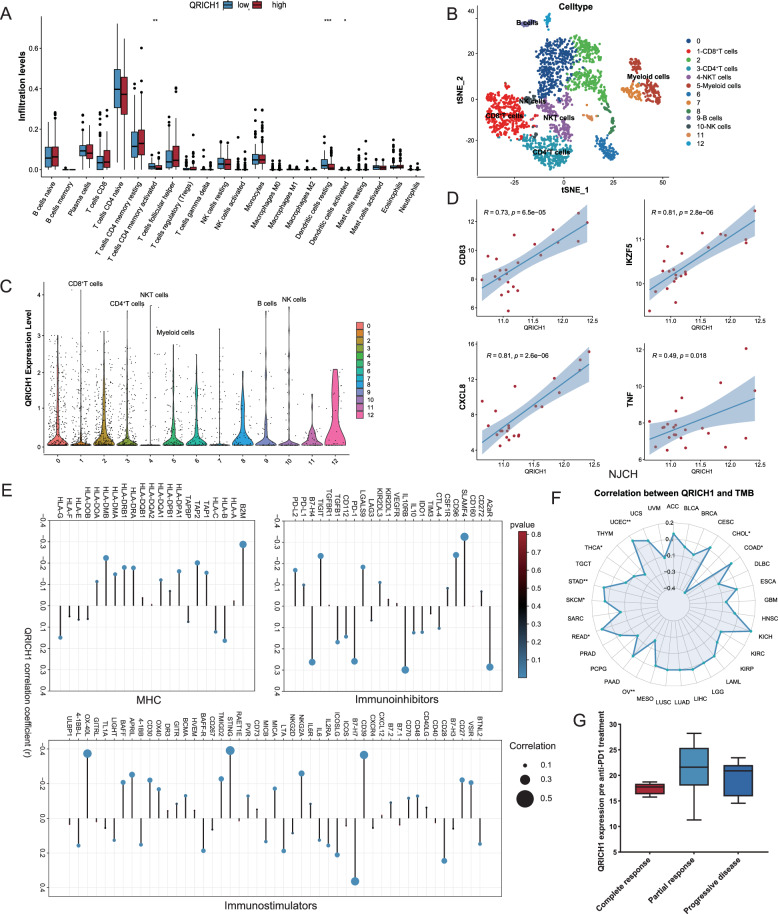
QRICH1 and tumor immunity. **A** CIBERSORT modeling of immune cell infiltration between high and low QRICH1 expression groups. **B** 13 clusters obtained after t-SNE downscaling of single-cell data from 5 normal donors and 5 refractory/relapsed T-ALL cases. **C** QRICH1 expression in 13 clusters. **D** Correlation analysis of QRICH1 with immune factors in T-ALL patients collected in our hospital. **E** Correlation analysis of QRICH1 with three categories of immunomodulatory factors. **F** Correlation between QRICH1 and TMB in the TCGA pan-cancer atlas. **G** QRICH1 expression stratified by PD-1 sensitivity.

Considering the role of GRP78 in favoring the pro-tumor immunity [27, 28], we further explored the immunomodulatory functions of QRICH1. Expression levels of QRICH1 changes with immune cell infiltration at varying degrees, especially “T cells CD4 memory resting” and “Dendritic cells” (Fig. 8A). We obtained 13 cell subsets after downscaling samples from 5 healthy donors and 5 refractory/relapsed T-ALL patients from the GSE161895 dataset (Fig. 8B). It is shown that the expression level of QRICH1 remained high in normal lymphocytes (Fig. 8C). In addition, there was a strong correlation of QRICH1 with multiple cytokines like CD83, IKZF5, CXCL8 and TNF in both T-ALL samples collected from our hospital and the TARGET-ALL-Phase II Project (Fig. 8D and Supplementary Fig. 2E). We further identified the correlation of QRICH1 with major histocompatibility complex (MHC) molecules and immunomodulators [29]. The expression level of QRICH1 was positively correlated with HLA-B and HLA-G, and negatively correlated with B2M, TAP1, TAP2, HLA-DPA1, HLA-DRA, HLA-DRB1, HLA-DMA, and HLA-DMB. Among immunoinhibitors, QRICH1 was positively correlated with A2aR, IL10RB, PD-1, CD112, TGFB1, and B7-H4, and negatively correlated with SLAMF4, CD96, LGALS9, TIGIT, and PD-L2. Correlation analysis with immunostimulators revealed that QRICH1 was positively correlated with BTNL2, CD28, B7-H7, ICOSLG, IL2RA, LTA, MICB, BAFF-R, 4-1BB, and 4-1BB-L, and negatively correlated with VSIR, CD27, CD39, NKG2A, MICA, STING, TMIGD2, OX40, CD30, APRIL, BAFF, and OX-40L (Fig. 8E). Correlated with both immunoinhibitors and immunostimulators indicated a dual role of QRICH1 in immunomodulation.

The tumor mutation burden (TMB) is a promising biomarker to predict the therapeutic efficacy of immune checkpoint inhibitors (ICIs) [30]. We found that the expression level of QRICH1 was correlated with TMB in multiple cancers in the TCGA datasets (Fig. 8F). In the GSE78220 dataset with 28 melanoma patients, the expression level of QRICH1 before PD-1 treatment was significantly lower in the complete response group compared to the partial response and progressive disease groups (Fig. 8G), supporting the immunomodulatory role of QRICHI in the tumor immunity.

## Discussion

T-ALL is featured as a poor prognosis in comparison to B-cell acute lymphoblastic leukemia (B-ALL). Although modern intensive chemotherapy protocols and allogeneic hematopoietic stem cell transplantation (HSCT) provide excellent survival benefits to high-risk T-ALL patients, some of them suffer from recurrences after experiencing a brief clinical remission [31]. Protein homeostasis is essential in maintaining cellular metabolism but is vulnerable to ERS attack by external and intrinsic factors. Eukaryotes are rapidly responsive to UPR as an adaptive cellular program [32]. However, this adaptive mechanism for maintaining protein homeostasis simultaneously promotes tumorigenesis and tumor progression [33]. After exceeding the threshold of ERS, an adaptive pattern of UPR fails to maintain the homeostasis and shifts to terminal UPR to cell death [34]. Currently, terminal UPR has been highlighted in oncology research [35].

We screened QRICH1 from a cluster of ERS-related genes and found its top three expressions in bone marrow, thymus, and testis, which led us to suspect that QRICH1 may function in hematopoiesis. Then, QRICH1 was identified as a candidate and significantly downregulated in pediatric T-ALL samples. Low QRICH1 expressions in T-ALL patients resulted in a poor prognosis, serving as an independent risk factor for the recurrence of pediatric T-ALL. In our center, patients’ data showed that QRICH1 was downregulated in high-risk subgroups of pediatric T-ALL. Collectively, we for the first time identified that QRICH1 may be a tumor-suppressor gene that predicted the prognosis of pediatric T-ALL.

In in vitro T-ALL models, overexpression of QRICH1 in Jurkat and HPBALL cells significantly inhibited cell proliferation. Consistently in the in vivo T-ALL mouse model, cell proliferation was also suppressed in mice overexpressing QRICH1. In T-ALL cells with QRICH1 overexpression, cells were mainly arrested in the G0/G1 phase, suggesting that QRICH1 inhibited cell proliferation by impairing DNA synthesis. However, controversial findings showed that the knockdown of QRICH1 in primary mouse epiphyseal chondrocytes does not influence cell proliferation, but chondrocyte differentiation is altered [36]. Our study also illustrated that overexpression of QRICH1 promoted apoptosis in T-ALL cells. In vivo experiments surprisingly revealed that overexpression of QRICH1 significantly prolonged the survival of T-ALL mice. Considering the survival benefit of QRICH1 in T-ALL as a load-shedding gene, we further deeply mined its biological functions. Both localized in the nucleus and cytoplasm, QRICH1 was broadly involved in RNA splicing and protein processing. We believed that QRICH1 shoulders the ultimate goal to increase intracellular protein burden, thereby causing proteotoxicity and terminal UPR [21], which was validated in our study.

GRP78, also known as immunoglobulin heavy chain binding protein (BiP), is an important chaperone protein within ER. It plays an important role in ER protein folding, modification, and secretion. In responsiveness to ER stress, GRP78 is responsible for signal transformation to the UPR to maintain homeostasis [23]. When GRP78 is inhibited, the three branches of UPR, namely the IRE1α, PERK, and ATF6 pathways, are persistently activated [37–40]. They further activate the downstream CHOP and trigger the cell death pathway [37, 38]. In our study, GRP78 was significantly downregulate by overexpressed QRICH1. Co-overexpression of GRP78 in QRICH1 partially reversed the regulatory effects of QRICHI on the proliferation and apoptosis of T-ALL cells, validating that GRP78 was essential for the anti-tumor role of QRICH1. LC-MS/MS and Co-IP confirmed that QRICH1 bound to GRP78 at Asp212 and Glu155 residues of NBD [24]. Interestingly, cMap data showed that most of compounds associated with high QRICH1 level were ATPase inhibitors. Therefore, we considered that QRICH1 acted as an ATPase inhibitor to bind to GRP78 and inhibit its function. In vitro findings also revealed a significant reduction in ATP content after overexpression of QRICH1, which may further affect the ATPase activity of GRP78.

Tm works as an inducer of ERS by blocking N-glycosylation modifications, thereby producing a large number of unfolded proteins and triggering UPR [41, 42]. QRICH1 is also known to cause terminal UPR. In our study, a persistent induction of Tm upregulated QRICH1 and increased cell death, suggesting that Tm may promote cancer cell death by activating QRICH1. Unfortunately, here we didn’t knock down QRICH1 for further rescue validation. It should be noted that in SYTO13 staining experiments, cell viability decreased not significantly at early ERS but significantly at 18–24h, which may be related to the fact that terminal UPR is more pronounced under sustained ERS [13]. The PERK-eIF2ɑ axis is responsible for regulating QRICH1 in necrotizing enterocolitis, combined with our research, there may be a complex network of loops involved in the regulation of QRICH1 [21, 43]. After overexpression of QRICH1 in prednisone-resistant HPBALL cells, drug resistance was effectively reversed, indicating the promising role of QRICH1 in chemotherapy resistance. We further searched QRICH1 gene mutations in the TCGA Pan-Cancer Atlas, and missense mutations were predominant. The mRNA expression of QRICH1 was also dominated by deletion expression. A previous neurodevelopmental study consistently reported that QRICH1 gene mutations are associated with defective expression and dysfunction [44].

GRP78 only partially rescued the phenotypic alterations in T-ALL cells overexpressing QRICH1. Considering the immunoregulatory role of UPR [45], we hypothesized that QRICH1 was also involved in it. Bioinformatic analysis showed that QRICH1 was correlated with immune cell infiltration, especially in T lymphocytes and dendritic cells. Single-cell sequencing showed that QRICH1 was upregulated in normal lymphocytes. In addition, QRICH1 was positively correlated with the expression levels of many immune factors, involved in dendritic cell differentiation, megakaryocyte maturation, and immune cell recruitment [46–49]. Furthermore, QRICH1 was identified to correlate with MHC molecules, immuneinhibitors and immunestimulators, serving as a dual-role regulator. Finally, low QRICH1 expression was predicted to have a high TMB, greater immunogenicity, and better response to ICI treatment. Future studies are needed to illustrate the role of QRICH1 in the tumor immunity of pediatric T-ALL.

## Conclusion

QRICH1 is a tumor-suppressor gene and prognostic biomarker of pediatric T-ALL. Overexpression of QRICH1 inhibits pediatric T-ALL by suppressing the downstream GRP78.

## Supplementary information

supplementary materials

## Data Availability

The relevant data for this study are in the manuscript and supplementary files, except for the 23 T-ALL data of our hospital, which are not publicly available at this time and can be obtained by contacting the corresponding author if reasonably necessary.
