# Supplementary material for: QRICH1 suppresses pediatric T-cell acute lymphoblastic leukemia by inhibiting GRP78
Source: Cell Death Dis. 2024 Sep 4;15(9):646. doi: 10.1038/s41419-024-07040-7 (PMC11371816; doi:10.1038/s41419-024-07040-7)
Supplement: Supplementary file 1 — supplementary materials [file 41419_2024_7040_MOESM1_ESM.pdf]

Supplementary Information 1

TARGET-ALL-Phase II Project

The inclusion criteria for cases:

1)T cell origin;

2)age at first diagnosis < 18 years old;

3)non-Down syndrome;

4)complete clinical data;

5) primary bone marrow sample, i.e., case number 17-24 "-09A-01R".

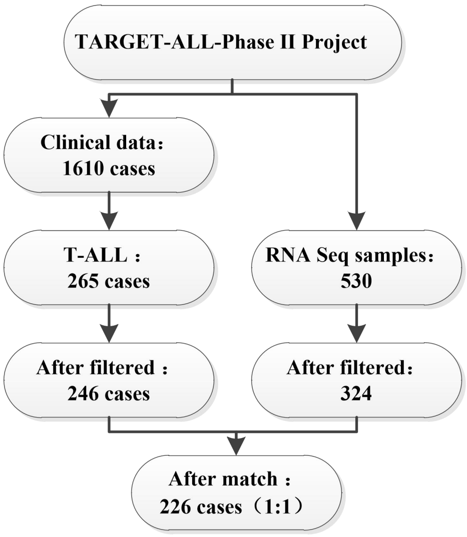

Supplementary Information 2

Sample ID	Cancer Type	Protein Change	Mutation Type	Start Pos	End Pos	Ref	Var	HGVSg
TCGA-55-A48Y-01	Non-Small Cell Lung Cancer	SETD2-QRICH1 Fusion	fusion	47163854				
TCGA-OR-A5LO-01	Adrenocortical Carcinoma	QRICH1-ACOT2 Fusion	fusion	49076709				
TCGA-G8-6909-01	Mature B-Cell Neoplasms	QRICH1-C3ORF62 Fusion	fusion	49032183				
TCGA-GC-A3RC-01	Bladder Cancer	QRICH1-PRKAR2A Fusion	fusion	49044390				
TCGA-13-1487-01	Ovarian Epithelial Tumor	QRICH1-MDGA1 Fusion	fusion	49093912				
TCGA-DD-AAD0-01	Hepatobiliary Cancer	QRICH1-TRAIP Fusion	fusion	49076709				
TCGA-KL-8336-01	Renal Non-Clear Cell Carcinoma	Q98E	Missense_Mutation	49114159	49114159	G	C	3:g.49114159G>C
TCGA-HT-8018-01	Glioma	K644E	Missense_Mutation	49070172	49070172	T	C	3:g.49070172T>C
TCGA-S9-A7QY-01	Glioma	X557_splice	Splice_Region	49083858	49083858	C	T	3:g.49083858C>T
TCGA-61-2003-01	Ovarian Epithelial Tumor	S278R	Missense_Mutation	49094799	49094799	A	C	3:g.49094799A>C
TCGA-30-1718-01	Ovarian Epithelial Tumor	A497D	Missense_Mutation	49084528	49084528	G	T	3:g.49084528G>T
TCGA-06-5416-01	Glioblastoma	N381K	Missense_Mutation	49094490	49094490	G	T	3:g.49094490G>T
TCGA-14-1458-01	Glioblastoma	S315P	Missense_Mutation	49094690	49094690	A	G	3:g.49094690A>G
TCGA-SG-A6Z7-01	Sarcoma	S618P	Missense_Mutation	49070596	49070596	A	G	3:g.49070596A>G
TCGA-22-1012-01	Non-Small Cell Lung Cancer	S773T	Missense_Mutation	49067898	49067898	C	G	3:g.49067898C>G
TCGA-18-3406-01	Non-Small Cell Lung Cancer	M389I	Missense_Mutation	49094466	49094466	C	G	3:g.49094466C>G
TCGA-77-8143-01	Non-Small Cell Lung Cancer	H329P	Missense_Mutation	49094647	49094647	T	G	3:g.49094647T>G
TCGA-85-A4QR-01	Non-Small Cell Lung Cancer	A520V	Missense_Mutation	49083970	49083970	G	A	3:g.49083970G>A
TCGA-98-8021-01	Non-Small Cell Lung Cancer	L156V	Missense_Mutation	49095167	49095167	G	C	3:g.49095167G>C
TCGA-37-4130-01	Non-Small Cell Lung Cancer	A197V	Missense_Mutation	49095043	49095043	G	A	3:g.49095043G>A
TCGA-37-4130-01	Non-Small Cell Lung Cancer	Q196L	Missense_Mutation	49095046	49095046	T	A	3:g.49095046T>A
TCGA-E5-A4TZ-01	Bladder Cancer	S4F	Missense_Mutation	49114440	49114440	G	A	3:g.49114440G>A
TCGA-DK-A3IT-01	Bladder Cancer	L379F	Missense_Mutation	49094498	49094498	G	A	3:g.49094498G>A
TCGA-DK-A3X1-01	Bladder Cancer	S261L	Missense_Mutation	49094851	49094851	G	A	3:g.49094851G>A
TCGA-4Z-AA7M-01	Bladder Cancer	S518F	Missense_Mutation	49083976	49083976	G	A	3:g.49083976G>A
TCGA-DK-AA6U-01	Bladder Cancer	S116L	Missense_Mutation	49095286	49095286	G	A	3:g.49095286G>A
TCGA-E7-A677-01	Bladder Cancer	N562S	Missense_Mutation	49081924	49081924	T	C	3:g.49081924T>C
TCGA-FD-A5C1-01	Bladder Cancer	L585V	Missense_Mutation	49081856	49081856	G	C	3:g.49081856G>C
TCGA-UY-A78K-01	Bladder Cancer	H126Y	Missense_Mutation	49095257	49095257	G	A	3:g.49095257G>A
TCGA-ZF-AA58-01	Bladder Cancer	E454K	Missense_Mutation	49084658	49084658	C	T	3:g.49084658C>T
TCGA-AB-2833-03	Leukemia	Q131H	Missense_Mutation	49095240	49095240	T	A	3:g.49095240T>A
TCGA-DD-AAC8-01	Hepatobiliary Cancer	R669W	Missense_Mutation	49070097	49070097	G	A	3:g.49070097G>A
TCGA-DD-AADP-01	Hepatobiliary Cancer	P658S	Missense_Mutation	49070130	49070130	G	A	3:g.49070130G>A
TCGA-CC-A7IK-01	Hepatobiliary Cancer	Q142L	Missense_Mutation	49095208	49095208	T	A	3:g.49095208T>A
TCGA-4R-AA8I-01	Hepatobiliary Cancer	L623M	Missense_Mutation	49070581	49070581	G	T	3:g.49070581G>T
TCGA-DD-AACL-01	Hepatobiliary Cancer	P695R	Missense_Mutation	49069670	49069670	G	C	3:g.49069670G>C
TCGA-EJ-AB20-01	Prostate Cancer	P264L	Missense_Mutation	49094842	49094842	G	A	3:g.49094842G>A
TCGA-XK-AAIW-01	Prostate Cancer	V413I	Missense_Mutation	49094396	49094396	C	T	3:g.49094396C>T
TCGA-KK-A7B3-01	Prostate Cancer	P702R	Missense_Mutation	49069649	49069649	G	C	3:g.49069649G>C
TCGA-AX-A3G8-01	Endometrial Cancer	D571Y	Missense_Mutation	49081898	49081898	C	A	3:g.49081898C>A
TCGA-EO-A22U-01	Endometrial Cancer	S472Y	Missense_Mutation	49084603	49084603	G	T	3:g.49084603G>T
TCGA-AP-A1E0-01	Endometrial Cancer	S162L	Missense_Mutation	49095148	49095148	G	A	3:g.49095148G>A
TCGA-B5-A0JY-01	Endometrial Cancer	P658S	Missense_Mutation	49070130	49070130	G	A	3:g.49070130G>A
TCGA-D1-A3DA-01	Endometrial Cancer	P121L	Missense_Mutation	49095271	49095271	G	A	3:g.49095271G>A
TCGA-AP-A054-01	Endometrial Cancer	K249R	Missense_Mutation	49094887	49094887	T	C	3:g.49094887T>C
TCGA-EY-A1GW-01	Endometrial Cancer	T88A	Missense_Mutation	49114189	49114189	T	C	3:g.49114189T>C

TCGA-DF-A2KU-01	Endometrial Cancer	R26M	Missense_Mutation	49114374	49114374	C	A	3:g.49114374C>A
TCGA-AP-A0LM-01	Endometrial Cancer	V174M	Missense_Mutation	49095113	49095113	C	T	3:g.49095113C>T
TCGA-AP-A059-01	Endometrial Cancer	V137A	Missense_Mutation	49095223	49095223	A	G	3:g.49095223A>G
TCGA-BS-A0TJ-01	Endometrial Cancer	A348V	Missense_Mutation	49094590	49094590	G	A	3:g.49094590G>A
TCGA-A5-A0G2-01	Endometrial Cancer	F628L	Missense_Mutation	49070566	49070566	A	G	3:g.49070566A>G
TCGA-A5-A0G2-01	Endometrial Cancer	E514*	Nonsense_Mutation	49083989	49083989	C	A	3:g.49083989C>A
TCGA-A5-A0G2-01	Endometrial Cancer	A19V	Missense_Mutation	49114395	49114395	G	A	3:g.49114395G>A
TCGA-A5-A1OF-01	Endometrial Cancer	D587G	Missense_Mutation	49081849	49081849	T	C	3:g.49081849T>C
TCGA-AJ-A3BG-01	Endometrial Cancer	T405M	Missense_Mutation	49094419	49094419	G	A	3:g.49094419G>A
TCGA-B5-A5OC-01	Endometrial Cancer	T405M	Missense_Mutation	49094419	49094419	G	A	3:g.49094419G>A
TCGA-AJ-A3BH-01	Endometrial Cancer	K612N	Missense_Mutation	49070612	49070612	C	A	3:g.49070612C>A
TCGA-AP-A059-01	Endometrial Cancer	X506_splice	Splice_Site	49084014	49084014	T	C	3:g.49084014T>C
TCGA-AP-A05N-01	Endometrial Cancer	P112L	Missense_Mutation	49095298	49095298	G	A	3:g.49095298G>A
TCGA-AP-A1DV-01	Endometrial Cancer	P698Q	Missense_Mutation	49069661	49069661	G	T	3:g.49069661G>T
TCGA-AP-A1DV-01	Endometrial Cancer	D65Y	Missense_Mutation	49114258	49114258	C	A	3:g.49114258C>A
TCGA-AX-A1CE-01	Endometrial Cancer	K557N	Missense_Mutation	49083858	49083858	C	A	3:g.49083858C>A
TCGA-AX-A1CE-01	Endometrial Cancer	A35P	Missense_Mutation	49114348	49114348	C	G	3:g.49114348C>G
TCGA-B5-A0JY-01	Endometrial Cancer	K656N	Missense_Mutation	49070134	49070134	C	A	3:g.49070134C>A
TCGA-B5-A1MR-01	Endometrial Cancer	R536Q	Missense_Mutation	49083922	49083922	C	T	3:g.49083922C>T
TCGA-B5-A3FA-01	Endometrial Cancer	R564M	Missense_Mutation	49081918	49081918	C	A	3:g.49081918C>A
TCGA-B5-A3FA-01	Endometrial Cancer	D417N	Missense_Mutation	49094384	49094384	C	T	3:g.49094384C>T
TCGA-B5-A3FA-01	Endometrial Cancer	X7_splice	Splice_Region	49114470	49114470	T	C	3:g.49114470T>C
TCGA-B5-A3FC-01	Endometrial Cancer	R533W	Missense_Mutation	49083932	49083932	G	A	3:g.49083932G>A
TCGA-B5-A3FC-01	Endometrial Cancer	S204Y	Missense_Mutation	49095022	49095022	G	T	3:g.49095022G>T
TCGA-EO-A22R-01	Endometrial Cancer	E29D	Missense_Mutation	49114364	49114364	T	G	3:g.49114364T>G
TCGA-EO-A22U-01	Endometrial Cancer	R234W	Missense_Mutation	49094933	49094933	G	A	3:g.49094933G>A
TCGA-EO-A22X-01	Endometrial Cancer	R230W	Missense_Mutation	49094945	49094945	G	A	3:g.49094945G>A
TCGA-EO-A3B0-01	Endometrial Cancer	K353T	Missense_Mutation	49094575	49094575	T	G	3:g.49094575T>G
TCGA-EO-A3KX-01	Endometrial Cancer	L726M	Missense_Mutation	49068040	49068040	G	T	3:g.49068040G>T
TCGA-EY-A548-01	Endometrial Cancer	A218V	Missense_Mutation	49094980	49094980	G	A	3:g.49094980G>A
TCGA-FI-A2D5-01	Endometrial Cancer	I330T	Missense_Mutation	49094644	49094644	A	G	3:g.49094644A>G
TCGA-67-6216-01	Non-Small Cell Lung Cancer	E184*	Nonsense_Mutation	49095083	49095083	C	A	3:g.49095083C>A
TCGA-38-7271-01	Non-Small Cell Lung Cancer	T308S	Missense_Mutation	49094711	49094711	T	A	3:g.49094711T>A
TCGA-55-8301-01	Non-Small Cell Lung Cancer	R511*	Nonsense_Mutation	49083998	49083998	G	A	3:g.49083998G>A
TCGA-17-Z022-01	Non-Small Cell Lung Cancer	G141D	Missense_Mutation	49095211	49095211	C	T	3:g.49095211C>T
TCGA-44-A479-01	Non-Small Cell Lung Cancer	A486S	Missense_Mutation	49084562	49084562	C	A	3:g.49084562C>A
TCGA-EB-A41A-01	Melanoma	E610K	Missense_Mutation	49070620	49070620	C	T	3:g.49070620C>T
TCGA-FS-A1ZK-06	Melanoma	S620F	Missense_Mutation	49070589	49070589	G	A	3:g.49070589G>A
TCGA-ER-A3PL-06	Melanoma	P161L	Missense_Mutation	49095151	49095151	G	A	3:g.49095151G>A
TCGA-FS-A4F5-06	Melanoma	K37*	Nonsense_Mutation	49114342	49114342	T	A	3:g.49114342T>A
TCGA-D3-A3MV-06	Melanoma	P698S	Missense_Mutation	49069662	49069662	G	A	3:g.49069662G>A
TCGA-EB-A41A-01	Melanoma	Q196*	Nonsense_Mutation	49095047	49095047	G	A	3:g.49095047G>A
TCGA-EE-A2GS-06	Melanoma	C554*	Nonsense_Mutation	49083867	49083867	A	T	3:g.49083867A>T
TCGA-FS-A1ZM-06	Melanoma	I9*	Frame_Shift_Del	49114419	49114426	AAGGAGAT	-	3:g.49114419_49114426del
TCGA-FS-A1Z3-06	Melanoma	X713_splice	Splice_Site	49068078	49068078	C	T	3:g.49068078C>T
TCGA-3N-A9WB-06	Melanoma	S446L	Missense_Mutation	49094296	49094296	G	A	3:g.49094296G>A
TCGA-QB-AA9O-06	Melanoma	S278R	Missense_Mutation	49094799	49094799	A	C	3:g.49094799A>C

TCGA-WE-A8K5-06	Melanoma	A348V	Missense_Mutation	49094590	49094590	G	A	3:g.49094590G>A
TCGA-D3-A1Q3-06	Melanoma	P346H	Missense_Mutation	49094596	49094596	G	T	3:g.49094596G>T
TCGA-D3-A2JO-06	Melanoma	R761I	Missense_Mutation	49067934	49067934	C	A	3:g.49067934C>A
TCGA-D3-A8GI-06	Melanoma	L559H	Missense_Mutation	49081933	49081933	A	T	3:g.49081933A>T
TCGA-D3-A8GI-06	Melanoma	W416*	Nonsense_Mutation	49094385	49094385	C	T	3:g.49094385C>T
TCGA-EE-A3AG-06	Melanoma	G527W	Missense_Mutation	49083950	49083950	C	A	3:g.49083950C>A
TCGA-ER-A19B-06	Melanoma	A348S	Missense_Mutation	49094591	49094591	C	A	3:g.49094591C>A
TCGA-FS-A1ZP-06	Melanoma	G320V	Missense_Mutation	49094674	49094674	C	A	3:g.49094674C>A
TCGA-QB-AA9O-06	Melanoma	Y279N	Missense_Mutation	49094798	49094798	A	T	3:g.49094798A>T
TCGA-HF-A5NB-01	Esophagogastric Cancer	A410T	Missense_Mutation	49094405	49094405	C	T	3:g.49094405C>T
TCGA-BR-4184-01	Esophagogastric Cancer	P154S	Missense_Mutation	49095173	49095173	G	A	3:g.49095173G>A
TCGA-HU-A4GQ-01	Esophagogastric Cancer	R720Q	Missense_Mutation	49068057	49068057	C	T	3:g.49068057C>T
TCGA-BR-8078-01	Esophagogastric Cancer	A292V	Missense_Mutation	49094758	49094758	G	A	3:g.49094758G>A
TCGA-AC-A2FF-01	Breast Cancer	E184K	Missense_Mutation	49095083	49095083	C	T	3:g.49095083C>T
TCGA-AC-A23H-01	Breast Cancer	D571Y	Missense_Mutation	49081898	49081898	C	A	3:g.49081898C>A
TCGA-A2-A0YK-01	Breast Cancer	G527R	Missense_Mutation	49083950	49083950	C	T	3:g.49083950C>T
TCGA-A2-A0EQ-01	Breast Cancer	E605Q	Missense_Mutation	49070635	49070635	C	G	3:g.49070635C>G
TCGA-AC-A2FF-01	Breast Cancer	E188K	Missense_Mutation	49095071	49095071	C	T	3:g.49095071C>T
TCGA-EW-A2FV-01	Breast Cancer	N694Ifs*6	Frame_Shift_Del	49069673	49069673	T	-	3:g.49069673del
TCGA-IB-7651-01	Pancreatic Cancer	R507H	Missense_Mutation	49084009	49084009	C	T	3:g.49084009C>T
TCGA-DM-A1HB-01	Colorectal Cancer	V216M	Missense_Mutation	49094987	49094987	C	T	3:g.49094987C>T
TCGA-AA-3713-01	Colorectal Cancer	N735Tfs*20	Frame_Shift_Del	49068014	49068014	G	-	3:g.49068014del
TCGA-AA-3673-01	Colorectal Cancer	A35S	Missense_Mutation	49114348	49114348	C	A	3:g.49114348C>A
TCGA-AA-3966-01	Colorectal Cancer	L282S	Missense_Mutation	49094788	49094788	A	G	3:g.49094788A>G
TCGA-AA-3715-01	Colorectal Cancer	Y709C	Missense_Mutation	49069628	49069628	T	C	3:g.49069628T>C
TCGA-AA-A010-01	Colorectal Cancer	L559I	Missense_Mutation	49081934	49081934	G	T	3:g.49081934G>T
TCGA-AG-A002-01	Colorectal Cancer	L572I	Missense_Mutation	49081895	49081895	G	T	3:g.49081895G>T
TCGA-AG-A002-01	Colorectal Cancer	K489N	Missense_Mutation	49084551	49084551	C	A	3:g.49084551C>A
TCGA-AA-3811-01	Colorectal Cancer	Q276R	Missense_Mutation	49094806	49094806	T	C	3:g.49094806T>C
TCGA-AA-A02R-01	Colorectal Cancer	M687V	Missense_Mutation	49069695	49069695	T	C	3:g.49069695T>C
TCGA-AZ-4315-01	Colorectal Cancer	E480D	Missense_Mutation	49084578	49084578	T	G	3:g.49084578T>G
TCGA-D5-6530-01	Colorectal Cancer	Q421*	Nonsense_Mutation	49094372	49094372	G	A	3:g.49094372G>A
TCGA-D5-6927-01	Colorectal Cancer	T215A	Missense_Mutation	49094990	49094990	T	C	3:g.49094990T>C
TCGA-SX-A7SN-01	Renal Non-Clear Cell Carcinoma	Q43*	Nonsense_Mutation	49114324	49114324	G	A	3:g.49114324G>A
TCGA-P3-A6T0-01	Head and Neck Cancer	T666M	Missense_Mutation	49070105	49070105	G	A	3:g.49070105G>A
TCGA-VS-A9UC-01	Cervical Cancer	S446L	Missense_Mutation	49094296	49094296	G	A	3:g.49094296G>A
TCGA-C5-A8YR-01	Cervical Cancer	L282F	Missense_Mutation	49094787	49094787	C	G	3:g.49094787C>G
TCGA-EA-A410-01	Cervical Cancer	T238M	Missense_Mutation	49094920	49094920	G	A	3:g.49094920G>A
TCGA-EK-A2PG-01	Cervical Cancer	P465Q	Missense_Mutation	49084624	49084624	G	T	3:g.49084624G>T
TCGA-XE-A9SE-01	Seminoma	Y406F	Missense_Mutation	49094416	49094416	T	A	3:g.49094416T>A

Supplementary table 1 Patients Information

Cases	Gender	Age (years)	Diagnosis	Fusion gene	Treatment
Patient 1	Male	6.3	T-ALL	-	CCCG-ALL2020
Patient 2	Male	13.5	T-ALL	SIL::TAL1	CCCG-ALL2020
Patient 3	Male	13.0	T-ALL	ETV6::chr4	CCCG-ALL2020

T-ALL, T-cell acute lymphoblastic leukemia.

Supplementary table 2 Primer sequences used for RT-qPCR

Genes	Species	Forward primer (5’-3’)	Reverse primer (5′-3′)
QRICH1	Human	AACCCTATGACCCAGATGTGC	TAGCCACTCCGTGAACCGAAC
GRP78	Human	CGGTCTACTATGAAGCCCGTCCA	AGCTTCATCTGGGTTTATGCCAC
CHOP	Human	GGAAACAGAGTGGTCATTCCC	CTGCTTGAGCCGTTCATTCTC
GAPDH	Human	TGTGGGCATCAATGGATTTGG	ACACCATGTATTCCGGGTCAAT
Ki67	Mouse	CGCAGGAAGACTCGCAGTTT	CTGAATCTGCTAATGTCGCCAA
PCNA	Mouse	TTGCACGTATATGCCGAGACC	GGTGAACAGGCTCATTCATCTCT
GAPDH	Mouse	AGGTCGGTGTGAACGGATTTG	TGTAGACCATGTAGTTGAGGTCA

Supplementary table 3 Baseline comparison between the low QRICH1 and high QRICH1 expression groups before propensity score matching

Variable	Low QRICH1	High QRICH1	Statistical value	P value
**Gender**				
male	83	90	χ^2=1.208	0.272
female	30	23		
**Age**				
<1 year or ≥10 years	53	42	χ^2=2.197	0.138
≥1 year and <10 years	60	71		
**Initial WBC**				
<100×10^9/L	44	65	χ^2=7.815	0.005
≥100×10^9/L	69	48		
**CNSL/TL statue**				
Y	38	31	χ^2=1.116	0.291
N	74	82		
**Day 29 MRD**				
<0.01	72	72	χ^2=0.000	1.000
≥0.01	41	41		
**Molecular biological characteristics**				
Unfavorable	3	7	χ^2=1.674	0.196
Favorable/other	110	106		

WBC, white cell count; CNSL/TL, central nervous system leukemia/ testicular leukemia; MRD, measurable residual disease.

Supplementary table 4 Baseline comparison between the low QRICH1 and high QRICH1 expression groups after propensity score matching

Variable	Low QRICH1	High QRICH1	Statistical value	P value
**Gender**				
male	37	37	χ^2=0.000	1.000
female	3	3		
**Age**				
<1 year or ≥10 years	15	15	χ^2=0.000	1.000
≥1 year and <10 years	25	25		
**Initial WBC**				
$<100\times10^9$/L	20	20	χ^2=0.000	1.000
$\geq100\times10^9$/L	20	20		
**CNSL/TL statue**				
Y	9	9	χ^2=0.000	1.000
N	31	31		
**Day-29 MRD**				
<0.01	29	29	χ^2=0.000	1.000
≥0.01	11	11		
**Molecular biological characteristics**				
Unfavorable	1	1	χ^2=0.000	1.000
Favorable/other	39	39		

WBC, white cell count; CNSL/TL, central nervous system leukemia/ testicular leukemia; MRD, measurable residual disease.

Supplementary table 5 Cox regression analysis of risk factors for the poor prognosis of pediatric T-ALL.

Variable	Partial regression coefficient	Standard error	Wald χ^2 value	p value	RR value
QRICH1	2.122	1.062	3.993	0.046	8.348

RR, relative risk.

Model evaluation: χ^2=5.713, p<0.05.

Supplementary table 6 Connectivity mapping of QRICH1

ID	Name	Type	Description	Norm CS
BRD-A67788537	salermide	CP	SIRT inhibitor	2.1603
BRD-K33277808	ouabain	CP	Na/K-ATPase inhibitor	2.1561
BRD-A34806832	proscillaridin	CP	Na/K-ATPase inhibitor	2.1494
BRD-A80502530	cinobufagin	CP	Na/K-ATPase inhibitor	2.1074
BRD-K06792661	narciclasine	CP	LIM kinase activator|Rho associated kinase activator	2.1068
BRD-A93236127	digitoxin	CP	Na/K-ATPase inhibitor	2.0939
BRD-A80502530	cinobufagin	CP	Na/K-ATPase inhibitor	2.0864
BRD-K06792661	narciclasine	CP	LIM kinase activator|Rho associated kinase activator	2.0793
BRD-K63606607	bufalin	CP	Na/K-ATPase inhibitor	2.0772
BRD-A34806832	proscillaridin	CP	Na/K-ATPase inhibitor	2.0737
BRD-K63606607	bufalin	CP	Na/K-ATPase inhibitor	2.0564
BRD-A45333398	periplocymarin	CP	Apoptosis stimulant	2.0302
BRD-K18518344	digitoxigenin	CP	Na/K-ATPase inhibitor	2.0237
BRD-A34806832	proscillaridin	CP	Na/K-ATPase inhibitor	2.022
BRD-A68930007	ouabain	CP	Na/K-ATPase inhibitor	2.0156
BRD-A67788537	salermide	CP	SIRT inhibitor	2.0125
BRD-K25504083	cytochalasin-d	CP	Tubulin inhibitor	2.0106
BRD-K07736136	VX-702	CP	P38 MAPK inhibitor	2.0093

CP, compound; Norm CS, the connectivity score.

A positive higher score means more positive connection between the bioactive compound and QRICH1.

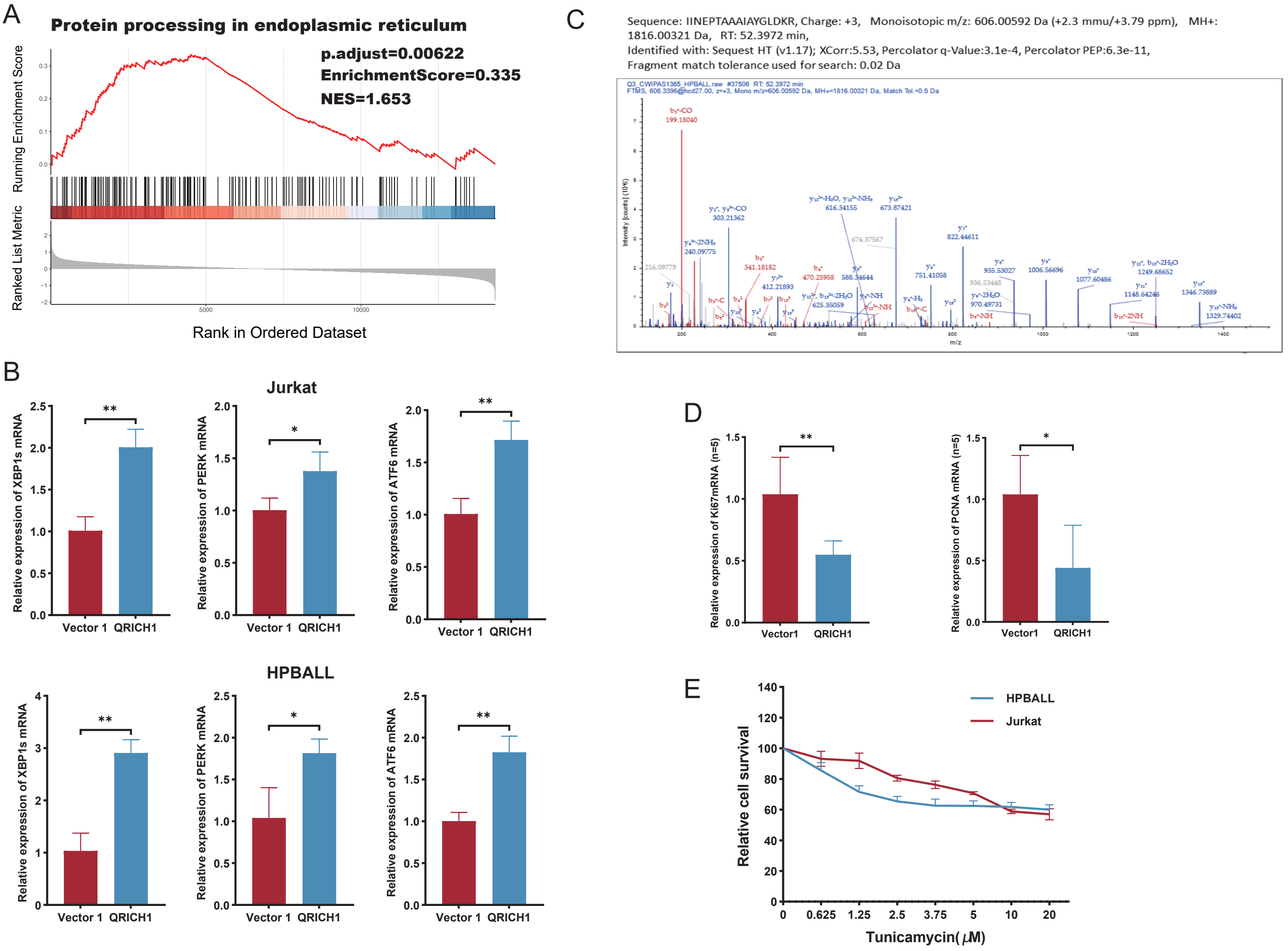

Supplementory Fig.1: **(A)**GSEA enrichment analysis revealed that QRICH1 is involved in the processing of endoplasmic reticulum proteins.**(B)**The mRNA expression of UPU markers XBP1s, PERK and ATF6 was significantly elevated in QRICH1 overexpressing cells.**(C)**LC-MS/MS analysis showed peptides corresponding to the qrich1-binding protein GRP78 in HPBALL cells.**(D)**Ki67 and PCNA mRNA expression in mice in the QRICH1 overexpression group was significantly lower than that in the vector group (n=5).**(E)**Jurkat and HPBALL cell viability at different Tm concentrations.

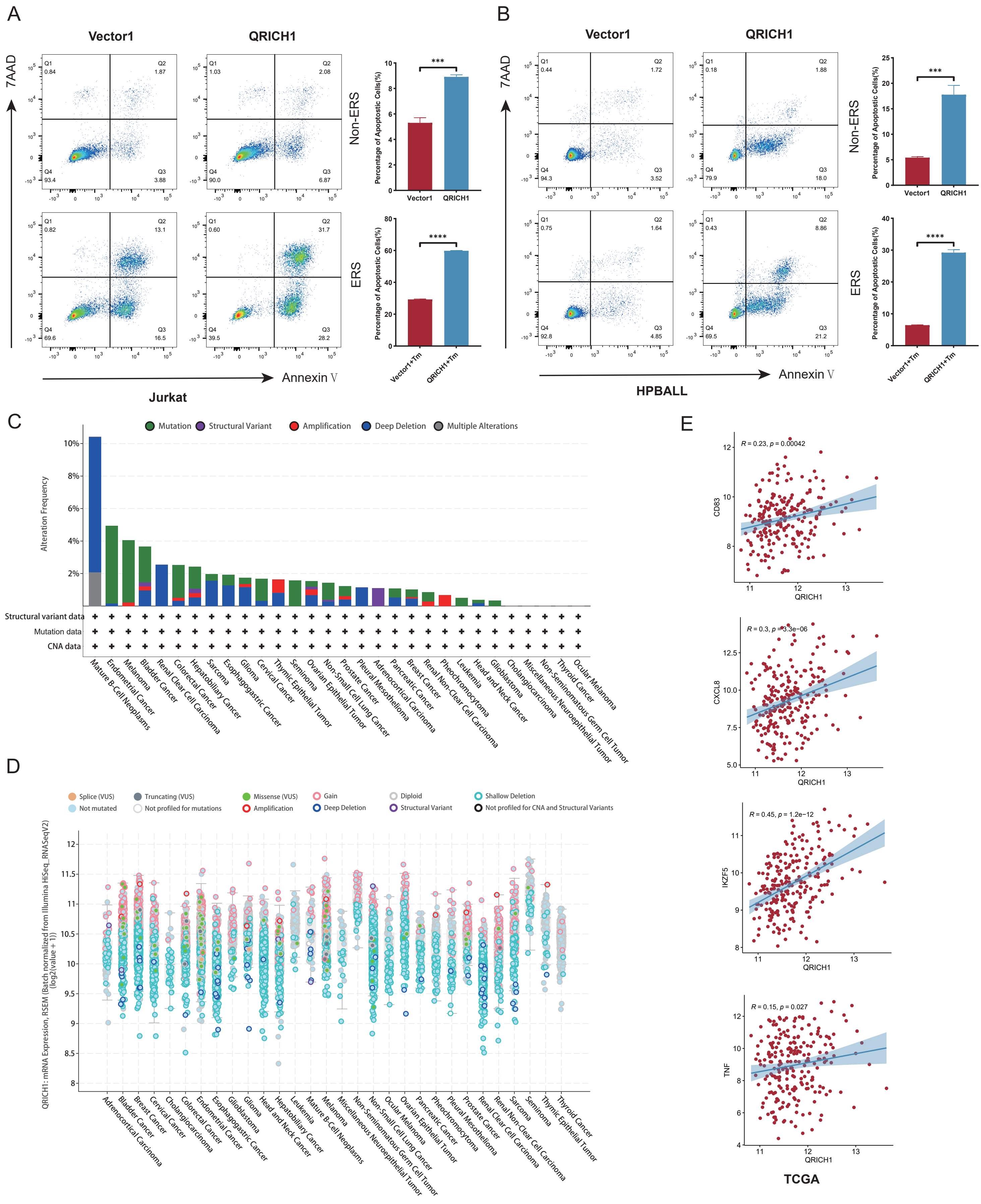

Supplementory Fig.2: **(A)**Jurkat cell apoptosis after QRICH1 overexpression in the absence and presence of ERS, respectively.**(B)**HPBALL cell apoptosis after QRICH1 overexpression in the absence and presence of ERS, respectively.**(C)**Bar chart of QRICH1mutations in 32 cancer studies based on TCGA PanCancer Atlas Studies.**(D)**Mutation counts and types of QRICH1 in pan-cancer.**(E)**Correlation analysis of QRICH1 and immunomodulatory molecules in the TCGA database.

qPCR original data

**Fig1.C**						
	CT	β-actin	▲CT	mean	▲▲CT	Q
Control	22.15666667	15.56333333	6.593333333	6.422222222	0.171111111	0.88815839
	21.98	15.72666667	6.253333333	6.422222222	-0.168888889	1.124192339
	22.12666667	15.70666667	6.42	6.422222222	-0.002222222	1.001541514
293T	16.42333333	10.29	6.133333333	6.422222222	-0.288888889	1.221699007
	16.41	10.31333333	6.096666667	6.422222222	-0.325555556	1.25314691
	16.57	10.33666667	6.233333333	6.422222222	-0.188888889	1.139885479
JUR	18.47333333	11.06666667	7.406666667	6.422222222	0.984444444	0.505420314
	18.52666667	11.12333333	7.403333333	6.422222222	0.981111111	0.506589433
	18.51666667	11.14333333	7.373333333	6.422222222	0.951111111	0.517233954
HPB	18.29333333	10.22	8.073333333	6.422222222	1.651111111	0.318394846
	18.36	10.35333333	8.006666667	6.422222222	1.584444444	0.333453051
	18.36666667	10.28	8.086666667	6.422222222	1.664444444	0.315465809
**Fig4.A**						Jurkat
CHOP	CT	GADGH	▲CT	mean	▲▲CT	Q
NC	25.66	18.46	7.2	7.24	-0.04	1.028113827
	25.5	18.41	7.09	7.24	-0.15	1.109569472
	25.79	18.5	7.29	7.24	0.05	0.965936329
	25.85	18.47	7.38	7.24	0.14	0.907519155
OE	26.18	19.1	7.08	7.24	-0.16	1.117287138
	25.87	19.11	6.76	7.24	-0.48	1.394743666
	25.85	19.08	6.77	7.24	-0.47	1.385109468
	25.93	19.29	6.64	7.24	-0.6	1.515716567
						HPBALLL
CHOP	CT	GAPDH	▲CT	mean	▲▲CT	Q
NC	21.1	16.64	4.46	4.6125	-0.1525	1.111493876
	21.26	16.4	4.86	4.6125	0.2475	0.842354841
	20.84	16.28	4.56	4.6125	-0.0525	1.037060457
	20.89	16.32	4.57	4.6125	-0.0425	1.029896957
OE	19.56	15.58	3.98	4.6125	-0.6325	1.550249044
	19.71	15.66	4.05	4.6125	-0.5625	1.476826146
	19.54	15.5	4.04	4.6125	-0.5725	1.487098284
	19.36	15.62	3.74	4.6125	-0.8725	1.830832745
**Fig4.C**						Jurkat
GRP78	CT	GAPDH	▲CT	mean	▲▲CT	Q
NC1	22.09	17.86	4.23	4.37	-0.14	1.101905116
	21.98	17.5	4.48	4.37	0.11	0.926588062
	21.94	17.45	4.49	4.37	0.12	0.920187651
	21.89	17.61	4.28	4.37	-0.09	1.064370182
OE1	22.41	17.79	4.62	4.37	0.25	0.840896415
	22.6	17.68	4.92	4.37	0.55	0.683020128
	22.54	17.55	4.99	4.37	0.62	0.650670928
	22.47	17.74	4.73	4.37	0.36	0.77916458
						HPBALL
GRP78	CT	GAPDH	▲CT	mean	▲▲CT	Q
NC	20.68	16.4	4.28	4.19	0.09	0.939522749
	20.54	16.28	4.26	4.19	0.07	0.952637998
	20.35	16.32	4.03	4.19	-0.16	1.117287138
OE	21.13	15.58	5.55	4.19	1.36	0.38958229
	21.07	15.56	5.51	4.19	1.32	0.400534939
	21.24	15.62	5.62	4.19	1.43	0.371130893

**Supplementory Fig1.B**						Jurkat
XBP1s	CT	β-actin	▲CT	mean	▲▲CT	Q
NC	20.20	10.03	10.17	9.893333333	0.276666667	0.825496117
	20.21	10.39	9.82	9.893333333	-0.073333333	1.052144848
	20.11	10.42	9.69	9.893333333	-0.203333333	1.15135548
OE	20.53	11.54	8.99	9.893333333	-0.903333333	1.870382496
	20.49	11.52	8.97	9.893333333	-0.923333333	1.896492062
	20.08	11.36	8.72	9.893333333	-1.173333333	2.255321854
PERK	CT	β-actin	▲CT	mean	▲▲CT	Q
NC	19.06	10.03	9.03	8.86	0.17	0.888842681
	19.24	10.39	8.85	8.86	-0.01	1.00695555
	19.12	10.42	8.7	8.86	-0.16	1.117287138
OE	19.99	11.54	8.45	8.86	-0.41	1.328685814
	20.09	11.52	8.57	8.86	-0.29	1.222640278
	19.56	11.36	8.2	8.86	-0.66	1.580082624
ATF6	CT	β-actin	▲CT	mean	▲▲CT	Q
NC	17.19	10.03	7.16	6.923333333	0.236666667	0.848703971
	17.27	10.39	6.88	6.923333333	-0.043333333	1.03049202
	17.15	10.42	6.73	6.923333333	-0.193333333	1.143402487
OE	17.60	11.54	6.06	6.923333333	-0.863333333	1.819236788
	17.85	11.52	6.33	6.923333333	-0.593333333	1.508728627
	17.42	11.36	6.06	6.923333333	-0.863333333	1.819236788
						HPBALL
XBP1s	CT	β-actin	▲CT	mean	▲▲CT	Q
NC	21.09	11.43	9.66	9.443333333	0.216666667	0.860551437
	21.15	11.41	9.74	9.443333333	0.296666667	0.814131268
	21.23	12.30	8.93	9.443333333	-0.513333333	1.427344254
OE	18.76	10.99	7.77	9.443333333	-1.673333333	3.189506754
	18.72	10.78	7.94	9.443333333	-1.503333333	2.834969734
	18.73	10.72	8.01	9.443333333	-1.433333333	2.700699892
PERK	CT	β-actin	▲CT	mean	▲▲CT	Q
NC	19.83	11.43	8.4	7.9	0.5	0.707106781
	18.80	11.41	7.39	7.9	-0.51	1.424050196
	20.21	12.30	7.91	7.9	0.01	0.993092495
OE	18.29	11.22	7.07	7.9	-0.83	1.777685362
	18.29	11.13	7.16	7.9	-0.74	1.670175839
	18.26	11.36	6.9	7.9	-1	2
ATF6	CT	β-actin	▲CT	mean	▲▲CT	Q
NC	17.57	10.99	6.58	6.743333333	-0.163333333	1.119871604
	17.57	10.78	6.79	6.743333333	0.046666667	0.968170696
	17.58	10.72	6.86	6.743333333	0.116666667	0.922316194
OE	17.06	11.22	5.84	6.743333333	-0.903333333	1.870382496
	17.18	11.13	6.05	6.743333333	-0.693333333	1.617015304
	17.11	11.36	5.75	6.743333333	-0.993333333	1.990779358
**Supplementory Fig1.D**						
Ki67	CT	GAPDH	▲CT	mean	▲▲CT	Q
NC	29.45666667	23.90666667	5.55	6.034666667	-0.484666667	1.39926253
	29.88666667	23.16666667	6.72	6.034666667	0.685333333	0.621862129
	29.23	23.22666667	6.003333333	6.034666667	-0.031333333	1.021956177
	27.95	21.78	6.17	6.034666667	0.135333333	0.910459448
	29.20666667	23.47666667	5.73	6.034666667	-0.304666667	1.235133228
OE	27.49333333	20.86333333	6.63	6.034666667	0.595333333	0.661891508

	30.29666667	23.14333333	7.153333333	6.034666667	1.118666667	0.460519239
	26.40333333	19.31	7.093333333	6.034666667	1.058666667	0.480075539
	30.11666667	23.52333333	6.593333333	6.034666667	0.558666667	0.678929338
	28.26333333	21.12	7.143333333	6.034666667	1.108666667	0.463722403
PCNA	CT	GAPDH	▲CT	mean	▲▲CT	Q
NC	27.40666667	23.90666667	3.5	4.026666667	-0.526666667	1.440596862
	27.28333333	23.16666667	4.116666667	4.026666667	0.09	0.939522749
	27.43	23.22666667	4.203333333	4.026666667	0.176666667	0.884744831
	26.42333333	21.78	4.643333333	4.026666667	0.616666667	0.652176035
	27.14666667	23.47666667	3.67	4.026666667	-0.356666667	1.280463977
OE	26.31666667	20.86333333	5.453333333	4.026666667	1.426666667	0.371989378
	27.12333333	23.14333333	3.98	4.026666667	-0.046666667	1.032875715
	26.24333333	19.31	6.933333333	4.026666667	2.906666667	0.13335403
	28.87666667	23.52333333	5.353333333	4.026666667	1.326666667	0.398688344
	27.06	21.12	5.94	4.026666667	1.913333333	0.265478451

Supplementary Material Original western blots images in manuscript

**Fig.1D** QRICH1	**Fig.1D** GAPDH	**Fig.1D** Complete
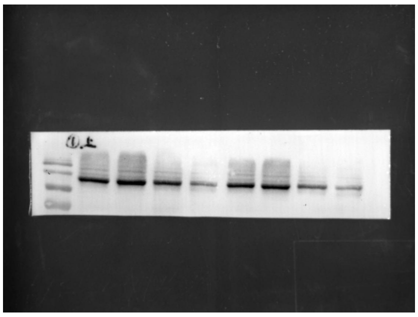	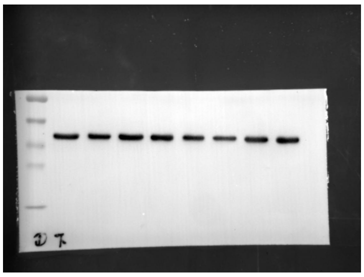	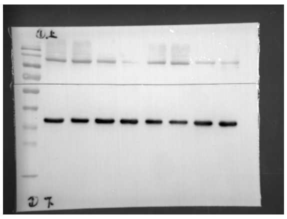
**Fig.1F** QRICH1	**Fig.1F** GAPDH	**Fig.1F** Complete
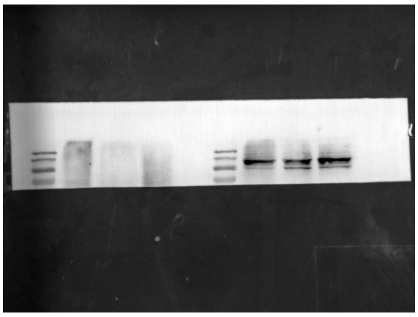	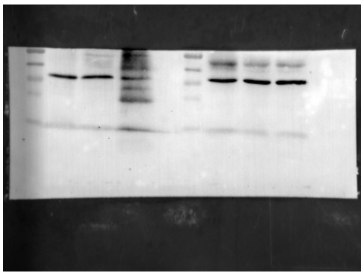	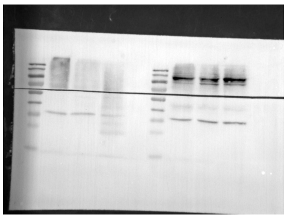
**Fig.2D** PARP (Jurkat)	**Fig.2D** cle-PARP (Jurkat)	**Fig.2D** cle-caspase9(Jurkat)
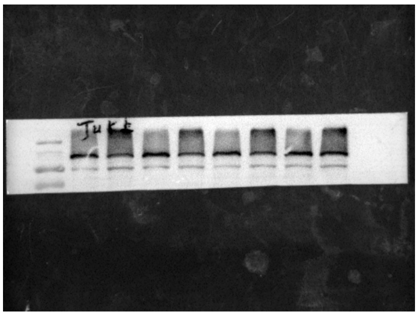	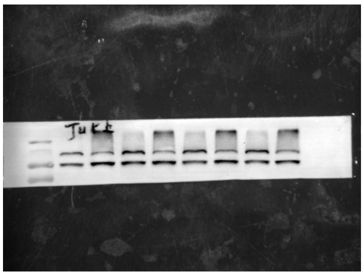	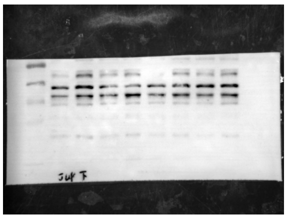
**Fig.2D** GAPDH (Jurkat)	**Fig.2D** PARP (HPBALL)	**Fig.2D** cle-PARP (HPBALL)
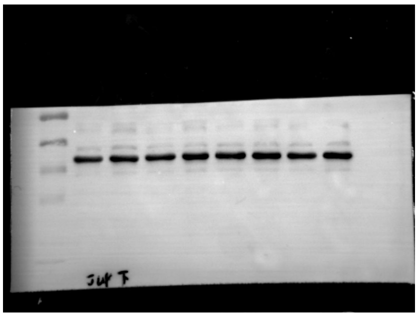	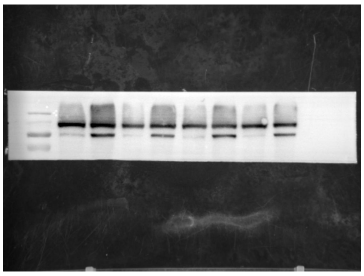	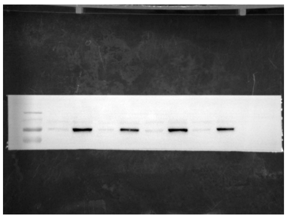
**Fig.2D** cle-caspase9(HPBALL)	**Fig.2D** GAPDH (HPBALL)	**Fig.2D** Complete

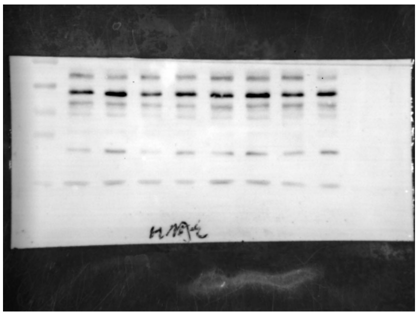	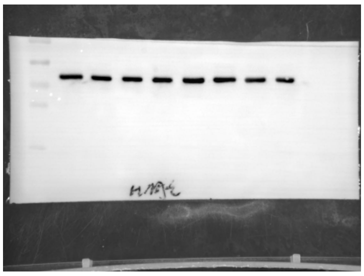	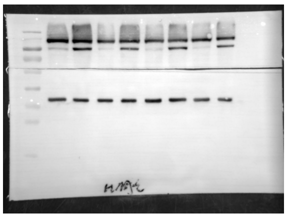
**Fig.4B** GRP78(Jurkat)	**Fig.4B** GAPDH(Jurkat)	**Fig.4B** GRP78(HPBALL)
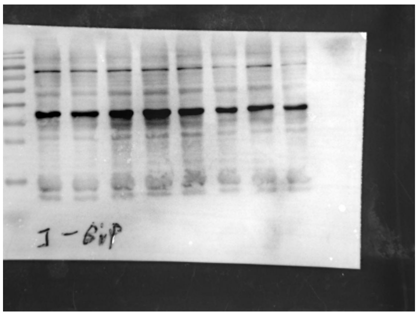	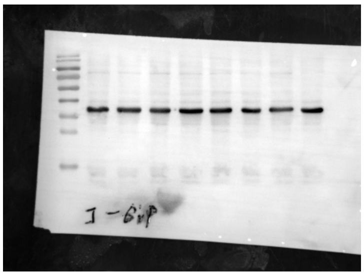	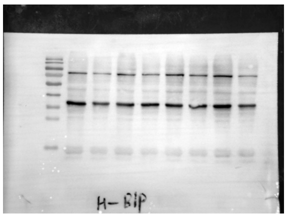
**Fig.4B** GAPDH(HPBALL)	**Fig.4D** CHOP(Jurkat)	**Fig.4D** GAPDH(Jurkat)
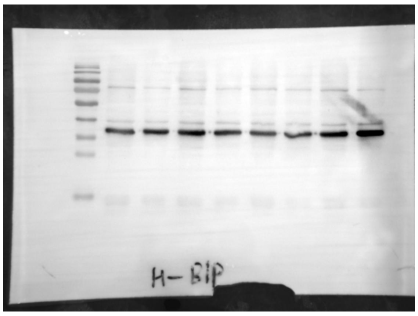	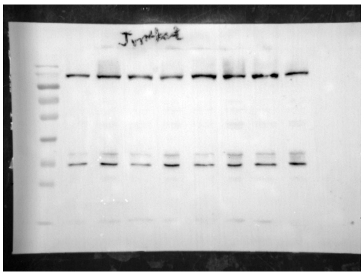	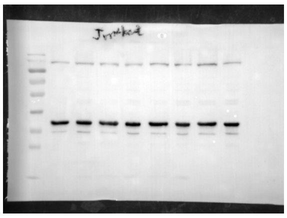
**Fig.4D** CHOP(HPBALL)	**Fig.4D** GAPDH(HPBALL)	
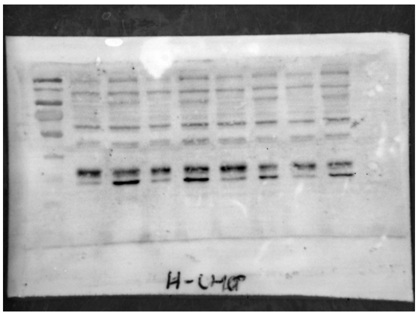	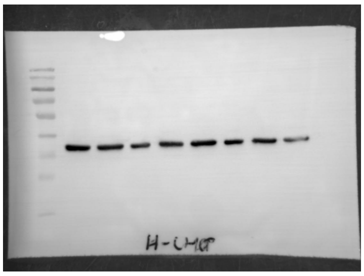	
**Fig.4E** cle-PARP(Jurkat)	**Fig.4E** cle-caspase9(Jurkat)	**Fig.4E** GAPDH(Jurkat)

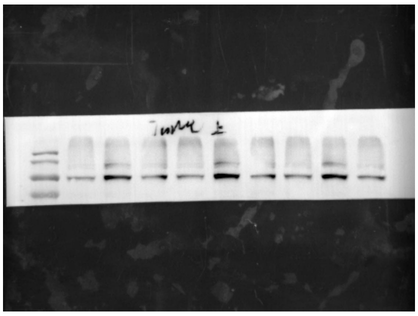	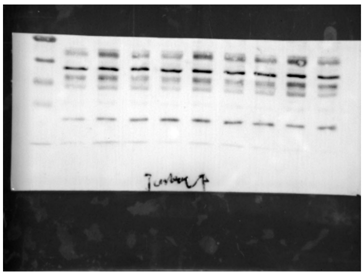	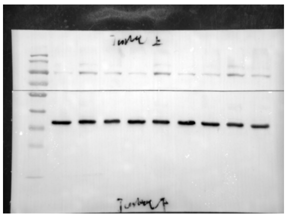
**Fig.4E** cle-PARP(HPBALL)	**Fig.4E** cle-caspase9(HPBALL)	**Fig.4E** GAPDH(HPBALL)
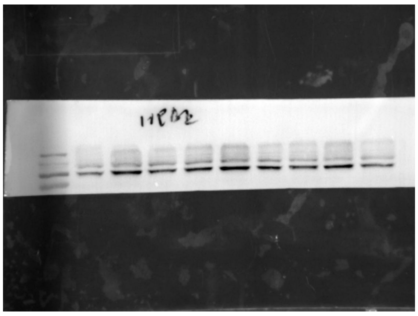	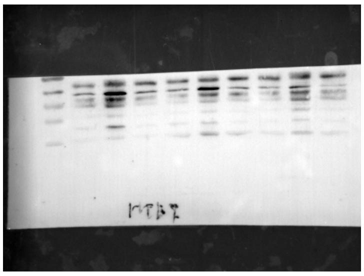	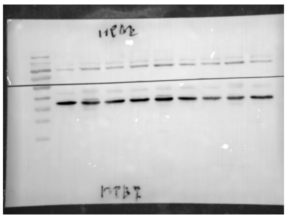
**Fig.5A** input FLAG (Jurkat)	**Fig.5A** input GRP78 (Jurkat)	**Fig.5A** IP FLAG (Jurkat)
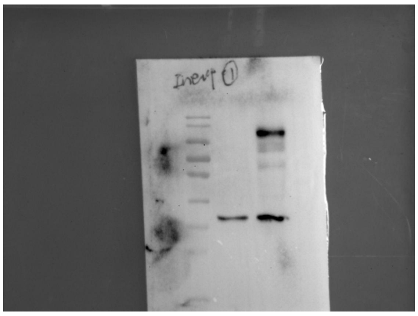	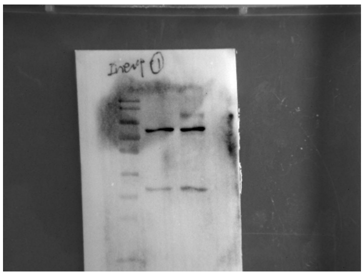	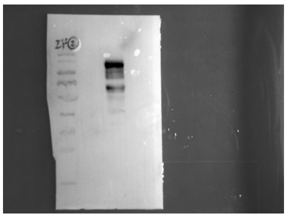
**Fig.5A** IP GRP78 (Jurkat)	**Fig.5A** input FLAG (HPBALL)	**Fig.5A** input GRP78 (HPBALL)
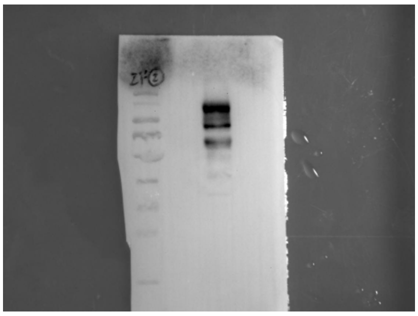	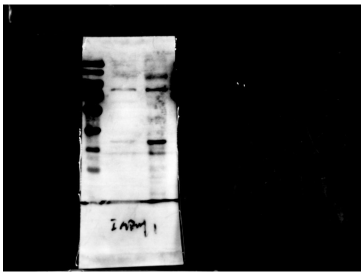	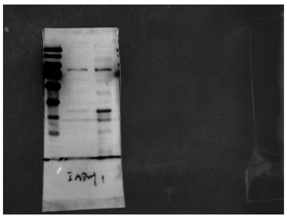
**Fig.5A** IP FLAG (HPBALL)	**Fig.5A** IP GRP78 (HPBALL)	

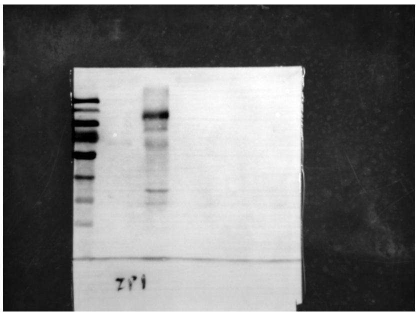	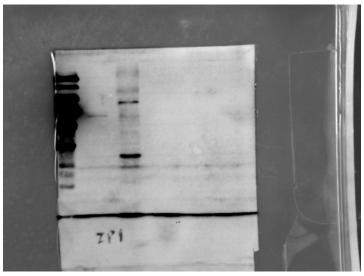	
**Fig.7C** QRICH1(Jurkat)	**Fig.7C** GAPDH(Jurkat)	**Fig.7C** Complete (Jurkat)
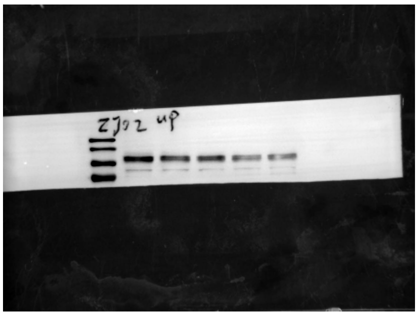	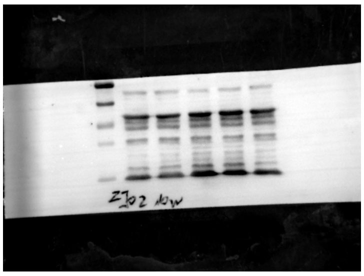	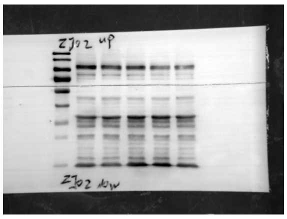
**Fig.7C** QRICH1(HPBALL)	**Fig.7C** GAPDH(HPBALL)	
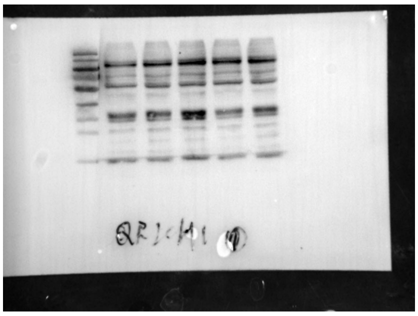	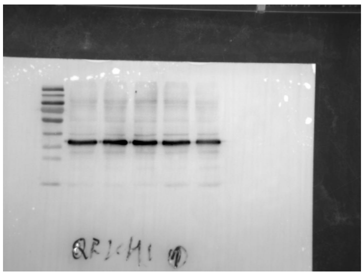	
